# Cenozoic climatic changes drive evolution and dispersal of coastal benthic foraminifera in the Southern Ocean

**DOI:** 10.1038/s41598-021-99155-6

**Published:** 2021-10-06

**Authors:** Wojciech Majewski, Maria Holzmann, Andrew J. Gooday, Aneta Majda, Tomasz Mamos, Jan Pawlowski

**Affiliations:** 1 Institute of Paleobiology, Polish Academy of Sciences, Twarda 51/55, 00-818 Warsaw, Poland; 2 Department of Genetics and Evolution, University of Geneva, Sciences III, 30 Quai Ernest Ansermet, 1211 Geneve 4, Switzerland; 3 National Oceanography Centre, European Way, Southampton, SO14 3ZH UK; 4 Department of Invertebrate Zoology and Hydrobiology, University of Lodz, Banacha 12/16, 90-237 Łódź, Poland; 5 Institute of Oceanology, Polish Academy of Sciences, Powstańców Warszawy 55, 81-712 Sopot, Poland

**Keywords:** Biogeography, Palaeontology, Phylogenetics, Population genetics, Taxonomy

## Abstract

The Antarctic coastal fauna is characterized by high endemism related to the progressive cooling of Antarctic waters and their isolation by the Antarctic Circumpolar Current. The origin of the Antarctic coastal fauna could involve either colonization from adjoining deep-sea areas or migration through the Drake Passage from sub-Antarctic areas. Here, we tested these hypotheses by comparing the morphology and genetics of benthic foraminifera collected from Antarctica, sub-Antarctic coastal settings in South Georgia, the Falkland Islands and Patagonian fjords. We analyzed four genera (*Cassidulina*, *Globocassidulina*, *Cassidulinoides*, *Ehrenbergina*) of the family Cassidulinidae that are represented by at least nine species in our samples. Focusing on the genera *Globocassidulina* and *Cassidulinoides*, our results showed that the first split between sub-Antarctic and Antarctic lineages took place during the mid-Miocene climate reorganization, probably about 20 to 17 million years ago (Ma). It was followed by a divergence between Antarctic species ~ 10 Ma, probably related to the cooling of deep water and vertical structuring of the water-column, as well as broadening and deepening of the continental shelf. The gene flow across the Drake Passage, as well as between South America and South Georgia, seems to have occurred from the Late Miocene to the Early Pliocene. It appears that climate warming during 7–5 Ma and the migration of the Polar Front breached biogeographic barriers and facilitated inter-species hybridization. The latest radiation coincided with glacial intensification (~ 2 Ma), which accelerated geographic fragmentation of populations, demographic changes, and genetic diversification in Antarctic species. Our results show that the evolution of Antarctic and sub-Antarctic coastal benthic foraminifera was linked to the tectonic and climatic history of the area, but their evolutionary response was not uniform and reflected species-specific ecological adaptations that influenced the dispersal patterns and biogeography of each species in different ways.

## Introduction

The Drake Passage and the Scotia Sea have a remarkable tectonic and geological history^1,2^ marked by major environmental and associated faunal changes^3^. The breakup of the supercontinent Gondwana started in the Mesozoic and continued through the Cenozoic. The last stage of this fragmentation was the opening of the Drake Passage between South America and West Antarctica, initiated at ~ 41 million years ago (Ma)^4^ or perhaps even earlier^5^. Progressive widening and deepening of this seaway enabled the formation of the Antarctic Circumpolar Current (ACC), which in combination with global carbon dioxide decline around the Eocene/Oligocene boundary ~ 34 Ma^6^, facilitated a gradual process of Antarctic cooling^7^, strengthening of thermal gradients between Patagonia and the Antarctic Peninsula, and a marked separation of ecosystems on the two sides of the Drake Passage^8^. This process reversed during the mid-Miocene climatic warming between 17 and 14.5 Ma, and even increased with the Middle Miocene cooling at ~ 14 Ma^9^ and the final opening of the Drake Passage for deep oceanic circulation ~ 12 Ma^10^.

In addition to these long-term changes, Antarctic organisms that inhabit the vast continental shelf have had to cope with repeated eradication and expansion events driven by ice-sheet dynamics. During the most severe glacial cycles, including the Last Glacial Maximum (LGM), most of the Antarctic continental shelf was covered by grounded ice sheets^11,12^, forcing benthic organisms into ice-free refugia^13–15^. Following deglaciation, the continental shelf was again available for re-colonization. Considering that at least 38 glacial cycles took place in Antarctica during the last 5 million years^16^, this mechanism may have acted as “the diversity pump” for marine benthos^17,18^. A similar process of recolonization of seafloor areas disturbed by ice-berg scouring^19^ and sediment gravity flows on the continental slope^13^ is currently taking place, although on a smaller scale.

This long and complex history of cooling, increased isolation, and repeated glaciations has profoundly shaped the Antarctic and Southern Ocean biota, resulting in many species being endemic to these regions^17,18,20–24^. However, the level of endemism may not be as extreme as often believed, perhaps closer to 50% than the figure 70–90% sometimes given^3^. This may reflect the breaching of the biogeographical barriers created by the ACC and the Polar Front (PF)^25^ during past interglacials and warm periods^17^, a process that has added further complexity to the community structure of Southern Ocean marine benthos.

Foraminifera are a dominant part of the Antarctic benthic meiofauna^26,27^. The Cassidulinidae, which are the focus of the present study, comprise a variety of species characterized by small calcareous hyaline tests. They are particularly abundant in polar regions, including the area on either side of the Drake Passage and the Scotia Sea^26–33^ considered in the present study. These protists are an important element of epicontinental benthic communities inhabiting areas impacted by Arctic^34^ and Antarctic glaciers^27,35,36^. As one of the key elements of the Antarctic fauna, the Cassidulinidae have been used extensively in paleoenvironmental research^35–39^ and are well represented in the Cenozoic fossil record from the Ross Sea^40–43^, the Antarctic Peninsula sector^44–47^, and East Antarctica^48–50^.

Despite their significance, the taxonomy of the Cassidulinidae remains poorly known due to difficulties in species distinction using morphological criteria^27^. Molecular phylogenetics can help with taxonomic determination, as has been shown by multiple studies^51–54^. However, only a few such studies have targeted Southern Ocean Cassidulinidae. These have included investigations of the morphological and molecular variability of *Globocassidulina biora* from Admiralty Bay in the South Shetlands^55^, and its biogeography in West Antarctica^56^. Differences have also been noted in population structure between Antarctic *G. biora* and its Patagonian sister species *Globocassidulina rossensis*^57^.

In the current study, we integrated morphological and genetic data for different species of Cassidulinidae from Antarctica and coastal areas of the Drake Passage and the Scotia Sea sector of the Southern Ocean. Our goal was to investigate the evolutionary processes that have led to their modern distribution and population structure, as well as to test the impact of geological and paleoclimatic events on the origin and dispersal patterns of coastal Southern Ocean foraminifera. The excellent fossil record of cassidulinids adds an additional perspective to addressing these objectives.

## Material and methods

### Sampling and laboratory analyses

**Figure 1:**
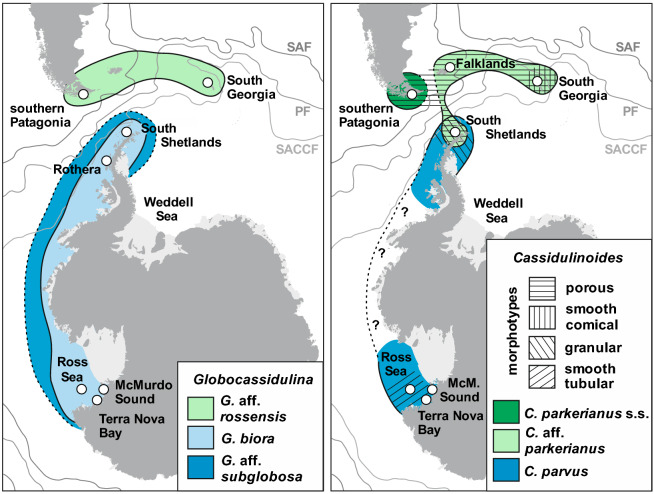
Maps showing sampling localities (white circles) and, in colour, schematically marked ranges of different species of *Globocassidulina* (left map) and *Cassidulinoides* (right map), based only on data presented in this paper. For *Cassidulinoides*, ranges of morphotypes are indicated by different hatching. Map data from https://freevectormaps.com. Location of the Subantarctic (SAF), Polar (PF), and southern Antarctic Circumpolar Current (SACCF) fronts after^25^.

Sampling locations are shown in Fig. 1. Sediment samples from the Ross Sea area were collected during shore-based operations mainly in McMurdo Sound during several field seasons between 1998 and 2011, as well as from the Ross Sea basins in 2015 during the Nathaniel B. Palmer RVIB expedition NBP1502. Samples from Admiralty Bay (King George Island, South Shetlands) were collected in early 2007 and from Rothera (Marguerite Bay) in 2013. Samples from north of the Drake Passage were collected from the Beagle Channel in 2007 and 2013, from South Georgia, and around Stanley (Falkland Islands) in late 2019. Sediment samples were obtained mainly with Van Veen and multicorer samplers as well as by divers. Locality information is provided in Appendix 1. Additional details on sample collection in Antarctica and Patagonia are given in^56^ and^58^, respectively.

Foraminiferal specimens were picked from the upper 2 cm of surface sediment samples. Sediment was washed, sieved and stored in cool (~ 4 °C) sea water. They were processed usually within a week of sampling. Residues > 125 μm, and occasionally also the 63–125 μm fraction, were scanned for foraminifera with visible cytoplasm. Individual specimens were transferred to guanidine lysis buffer or placed on micropaleontological slides and air dried. Sediment samples from Rothera were wet-sieved and frozen after collection, and the extraction of individual specimens took place later at the Department of Genetics and Evolution, University of Geneva. Residues were subsequently dried and foraminifera selected under a stereoscopic microscope. Specimens were arranged according to their morphology on micropaleontological slides. Typical specimens, potentially representing distinct species, were photographed using a Phillips XL20 Scanning Electron Microscope (SEM).

### Extraction, PCR, cloning and sequencing

In most cases, DNA was extracted from single individuals in a guanidine lysis buffer^59^. The 3′ fragment of the SSU rRNA gene was amplified with s14F3 (5′ACGCAMGTGTGAAACTTG)-sB (5′TGATCCTTCTGCAGGTTCACCTAC) and reamplified with 14F1 (5′AAGGGCACCACAAGAACGC)-sB. The fragment represents the standard barcoding fragment in foraminifera^52^. For some specimens, either primer newB (5′TGCCTTGTTCGACTTCTC) or primer 20r (5′GACGGGCGGTGTGTACAA) was used instead of sB.

The amplified PCR products were purified using High Pure PCR Cleanup Micro Kit (Sigma-Aldrich). Some PCR products were cloned with the TOPO TA Cloning Kit (Invitrogen), following the manufacturer's instructions and transformed into competent *E. coli*. Sequencing reactions were performed using the BigDye Terminator v3.1 Cycle Sequencing Kit (Thermo Fisher Scientific) and analyzed on a 3130XL Genetic Analyzer (Applied Biosystems). The new sequences reported in this paper were deposited in the EMBL/GenBank database; all access numbers are listed in Appendix 1.

### Species delimitation and divergence

Our genetic dataset for Cassidulinidae species includes 105 partial SSU rDNA sequences from Antarctica, 53 from southern Patagonia, 27 from South Georgia, and 14 from the Falkland Islands (Table 1 and Appendix 1). Of these 199 sequences, 160 sequences were obtained for this study. The 39 sequences of *Globocassidulina biora* and *Globocassidulina rossensis* have been published previously in^56^ and^57^. Sequences were manually aligned using SeaView v. 5.0.1 software^60^.

**Table 1:** Number of isolates/clones, genetic dissimilarity, and geographical distribution in different taxa of the Cassidulinidae family from the Southern Ocean.

Taxon	Material*	Genetic dissimilarity (%)	Geographical distribution**
*Globocassidulina* spp.	44/85	0–11.2	
*G. biora*	17/32	0–1.8	RS, ROT, ADM
*G.* aff. *rossensis*	11/20	0.2–10.8	PAT, SG
*G.* aff. *subglobosa*	16/32	0–2.3	RS, ROT, ADM
*Cassidulinoides* spp.	38/69	0–9.6	
*C. parvus*	12/26	0–3.9	RS, ADM
granular morphotype	5/8	0–0.6	ADM
smooth morphotype	7/18	0–1.3	RS
*C.* aff*. parkerianus*	16/21	0–3.8	SG, FK, ADM
Antarctic population	2/4	0–0.4	ADM
*C. parkerianus* s.s	9/22	0–1.9	PAT
*Cassidulina* spp.	25/37	0–15.8	PAT, SG, FK, ADM
*Ehrenbergina glabra*	3/9	0–1.1	RS

*Number of isolates (1st number) and clones (2nd number).

**Antarctica: *RS* Ross Sea, *WS* Weddell Sea, *ROT* Rothera, Margarite Bay, *ADM* Admiralty Bay, South Shetlands, as well as *PAT* southern Patagonia, *FK* Falkland Islands, and *SG* South Georgia.

To identify the number of Molecular Operational Taxonomic Units (MOTUs) that could represent putative species, we applied four different methods: (1) barcode-gap approach using the Automatic Barcode Gap Discovery (ABGD) software^61^ and phylogeny based, (2) General Mixed Yule Coalescent (GMYC) model-based method^62^, (3) the Bayesian implementation of the Poison Tree Processor (bPTP)^63^, and (4) multi rate PTP (mPTP)^64^.

The ABGD method is based upon pairwise distance measures. We used primary partitions as a principal for group definition, as they are typically stable over a wider range of prior values, minimize the number of false positive (over-split species), and are usually close to the number of taxa described by taxonomists^61^. Default values from 0.001 to 0.1 were explored as intraspecific distances and gap values from 1 to 1.5. The standard Jukes-Cantor model correction was applied. The ABGD analyses was performed on the entire dataset (step 1) as well as on subsets of sequences representing four distinct genera (step 2). Results of these analyses are provided in Appendix 2.

Because GMYC uses ultrametric trees, we applied BEAST 2.5.2^65^ to reconstruct Bayesian phylogeny for the input tree. All SSU haplotypes were used and no outgroup was present in the dataset. The site model and prior selection followed the methodology from time-calibrated reconstruction of phylogeny. Four runs of Markov chain Monte Carlo (MCMC) were performed, each 20 M generations long and sampled every 1,000 generations. Analysis was performed in the R software package `SPLITS' (Species Limits by Threshold Statistics)^66^ in R v3.1.0^67^.

The tree-based bPTP and mPTP methods use a priori generated phylogram. For the input tree, we used a Maximum likelihood (ML) tree obtained with RAxML^68^ from the haplotype data. The ML analysis was run under a thorough tree search with the model parameter set to GTRGAMMAI. The bPTP analysis was performed on the bPTP web server (available at http://www.species.h-its.org/ptp/) with 500,000 iterations of MCMC and 10% burn-in. Two runs were performed. The convergence of each run was verified by a stationary pattern of the MCMC iterations trace plot. Both runs provided congruent results. The mPTP method implements MCMC sampling that provides fast and comprehensive evaluation of the inferred delimitation. Five runs of 100 M MCMC generations long chain with burn in of 10% were performed.

To account for insertions/deletions in variable regions of the SSU rRNA gene, the MOTUs reflecting division at the species level were further validated using sequence divergences, calculated with BioEdit v 7.2^69^. Relationships between different haplotypes were illustrated with a haplotype network, generated using default parameters set up for the popart 1.7 software^70^ and the Median-Joining algorithm^71^. Separate haplotype matrices were prepared manually including all mutations and insertions/deletions as single events, regardless of their length, in order to get a detailed visualization of population structure of MOTUs/species.

### Demography

In order to assess putative demographic changes in populations of the analyzed species, two approaches were employed. Firstly, neutrality tests, including Tajima's D^72^ and Fu's Fs^73^, were calculated using DNASP^74^. Their statistical significance was evaluated using coalescent simulations with 1000 replications. Secondly, the extended Bayesian skyline plot (eBSP)^75^ in BEAST 2.5.2^65^ was employed to visualize the demographic changes through time. As the prior clock rate, we used the mutation rate obtained from the time calibrated phylogeny reconstruction (mean: 0.00345, SD: 0.000040615). The MCMC chain was run two times to ensure convergence for 20 million generations, sampled every 10 000 generations. One run for each data set was used to plot the eBSP in R package (http://www.r-project.org) after a 10% burn-in phase.

### Time calibrated phylogeny reconstruction

To estimate the temporal framework of the evolutionary history of Antarctic Cassidulinidae, we reconstructed the time-calibrated tree using a SSU rDNA reduced data set in BEAST 2.5.2^65^. For the reduced data set, only selected sequences representing central haplotypes in networks were used. Two calibration points were applied based on previous studies^76,77^. The two nodes are the divergence between *Uvigerina peregrina* (seq. AY914574) and *Trifarina earlandi* dated at 33.9 Ma (nod A in Fig. 7), and between *Cibicides ungerianus* (seq. FJ705898) and *Cibicides wuellerstorfi* (seq. AY934741) dated at 15 Ma (nod B in Fig. 7). Both calibration points were tested in a previous study^57^ and represent robustly dated events, where the fossil record agrees with published molecular phylogenetic reconstructions^78^. The substitution model was selected via bModelTest^79^. Birth–Death tree model (> 3BF) and strict clock (> 20BF) were set as priors through path sampling selection. Four runs of the MCMC, each 10 million generations long and sampled every 1000 generations, were performed and examined for convergence in TRACER 1.7^80^. All runs reached the effective sample size (ESS) above 200 and were combined using LogCombiner 2.5.2^65^. The final maximum clade credibility tree was summarized with TreeAnnotator 2.5.2^65^. In order to provide bootstrap supports for the phylogeny, the Maximum Likelihood (ML) approach was applied through RAxML 8.2.8^68^. The best-scoring ML trees were produced using the GTRGAMMAI parameter. Bipartition information was drawn from 10 run replicates, statistical supports were estimated with thorough bootstrap tests set to 1000 repetitions.

To validate the time calibration, we used two additional calibration points set within the Cassidulinidae, one based on the divergence of *Cassidulina* and *Globocassidulina,* the other on the divergence of *Ehrenbergina* and *Cassidulinoides*. Cassidulinidae are present in the fossil record at least since the Paleocene^81^. Because fossil specimens similar to *G. subglobosa* are known from the earliest Eocene of the NE Atlantic^82^ and West Antarctica^45^, and *Cassidulina* was recorded in Peru at least since Middle Eocene^83^, their divergence should have taken place between the Paleocene-Eocene benthic foraminiferal turnover, coinciding with the Paleocene-Eocene Thermal Maximum at ~ 56 Ma^84^, and ~ 45 Ma. Concerning the second divergence, the oldest fossils of *Ehrenbergina* were described from the upper Middle Eocene of Italy^85^, while *Cassidulinoides* was reported from the Upper Eocene of Alabama^86^. Thus, the split between *Ehrenbergina* and *Cassidulinoides* occurred most likely between ~ 56 Ma and ~ 38 Ma. These ranges suggested by the fossil record are indicated on the phylogenetic tree.

## Results

### Integrative taxonomy

Results of the ABGD analysis (step 1) revealed four groups (Appendix 2). The results are stable between P = 0.0026 and P = 0.0183. These are consistent with four Cassidulinidae genera, namely *Globocassidulina, Cassidulina*, *Cassidulinoides* and *Ehrenbergina*, which are also well characterized morphologically^81^. The genera *Globocassidulina* and *Cassidulina* have biserially arranged chambers that are planispirally coiled in both juvenile and mature individuals (Appendix 3). In species of *Globocassidulina,* the tests are sub-spherical. Species of *Cassidulina* include forms with lenticular tests that were assigned initially to *C. laevigata* and *C. carinata*, as well as forms with moderately inflated tests assigned initially to *C. crassa* and *C. minuta.* Representatives of *Cassidulinoides* and *Ehrenbergina* have biserial tests that are enrolled only in the first whorls (Appendix 3). Adult, uncoiled chambers of the later are clearly flattened dorso-ventrally and armed with lateral spines.

**Figure 2:**
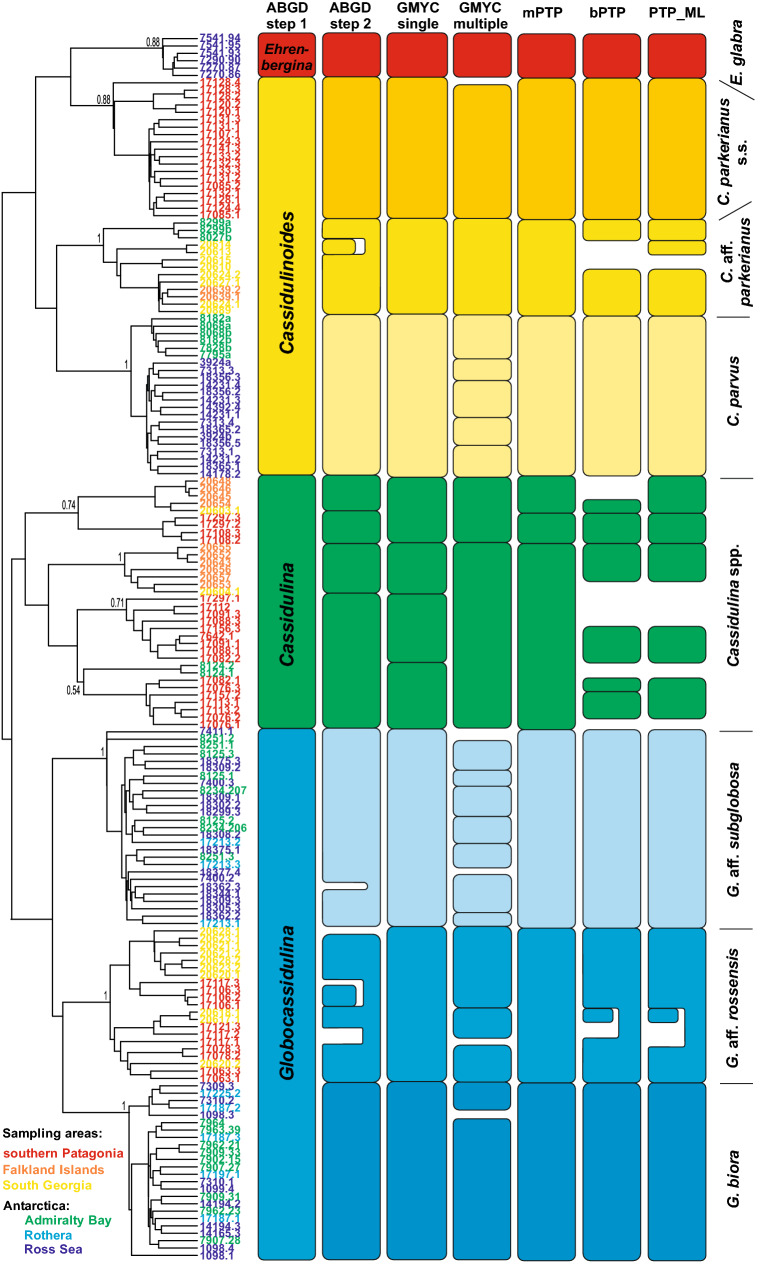
Molecular operational taxonomical units (MOTUs) or species, marked by shades of different colors, based on six different species delimitation analyses. MOTUs with single sequences not indicated. Bayesian ultrametric tree is based on haplotype data reconstructed in BEAST 2.5. Sequence numbers are colored according to sampling location.

Tree-based delimitation analyses and step 2 of the ABGD analysis suggested the presence of several MOTUs/species within each genus except *Ehrenbergina* (Fig. 2 and Appendix 2). In both GMYC methods, the null model of a single coalescent species was rejected (P < 0.0001). The single threshold model-based GMYC revealed 11 MOTUs and the multiple-threshold model 28 MOTUs, while the mPTP method resulted in 10 MOTUs and the bPTP and ML methods in 27–32 MOTUs.

Genetically, all genera (except *Ehrenbergina*) show high variability. The greatest genetic dissimilarity, surpassing 15%, was found in *Cassidulina* (Table 1). Each of the six methods for delimiting species suggested different numbers of MOTUs/species (Fig. 2). It was also not uncommon to find different ribotypes, i.e. sequences obtained from a single isolate (e.g., isolates 17,076, 17,082, and 17,297), attributed to different MOTUs. Sequences were obtained from 26 isolates (Appendix 1), although only a single isolate (8124) originated from Antarctica. Based on the large morphological variability (Appendix 3) and heterogeneous molecular results, it seems likely that several species of *Cassidulina* are present in the Southern Ocean, although our current dataset is too limited to identify or describe them. Based on delimitation analyses, one species of *Ehrenbergina* was identified. Our dataset comprises sequences obtained from three isolates collected in shallow water settings of the Ross Sea, Antarctica (Appendix 1), the area from which *E. glabra* was originally described^87^.

**Figure 3:**
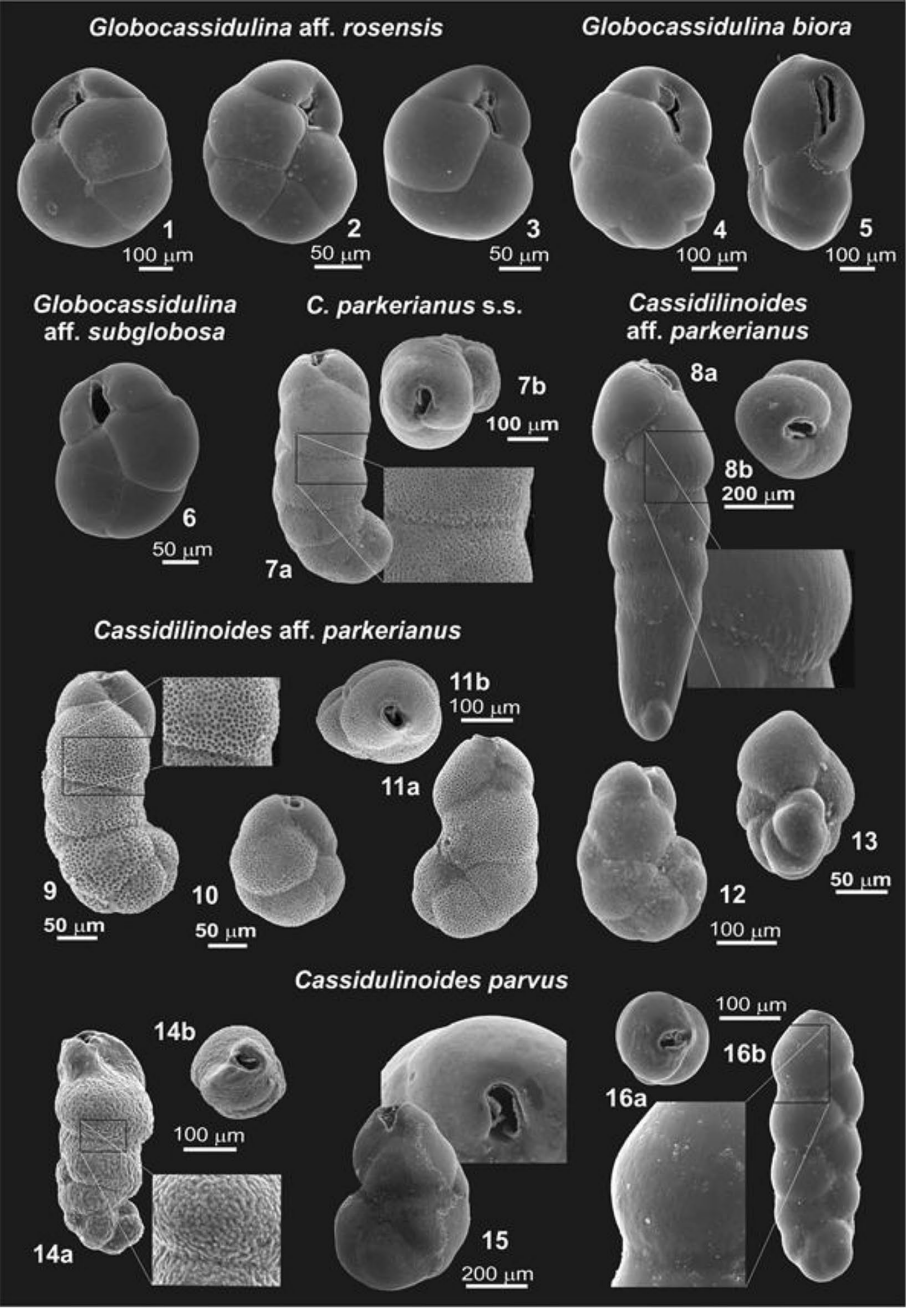
SEM images of Cassidulinidae from the Drake Passage and the Scotia Sea sector of South Atlantic, including West Antarctica: (**1**–**3**) *Globocassidulina* aff. *rossensis* from southern Patagonia, the Falklands, and South Georgia; (**4–5**) *Globocassidulina biora* from South Shetlands, immature form with bifurcated aperture and mature one with doubled aperture; (**6**) *Globocassidulina* aff. *subglobosa* from the Ross Sea; (**7**) *Cassidulinoides parkerianus* s.s. from southern Patagonia; (**8**, **13**) *Cassidulinoides* aff. *parkerianus*, the smoothly-walled conical morphotype from South Georgia; (**9–12**) *Cassidulinoides* aff. *parkerianus*, the porous morphotype from South Georgia (two specimenes), South Shetlands, and the Falklands; (**14–16**) *Cassidulinides parvus*, granular morphotype from South Shetlands, megalospheric and microsphelic variants of the smoothly-walled tubular morphotype from the Ross Sea. For more images showing full morphological diversity, see Appendix 3.

Our most comprehensive datasets were for species of *Globocassidulina* and *Cassidulinoides* (Fig. 3). Their nomenclature is provided in Appendix 4. For *Globocassidulina,* a majority of the molecular delimitation analyses indicated the presence of three species (Fig. 2) with well-defined geographical ranges; *G.* aff*. rossensis* limited to the sub-Antarctic, and *G. biora* and *G.* aff. *subglobosa,* limited to the Antarctic (Fig. 1). Large tests of *G. biora* and *G.* aff. *rossensis* have similar chamber arrangements and are moderately flattened [Fig. 3(1–5)]. Adult tests of *G. biora* display a double aperture [Fig. 3(5)], but some immature specimens have a bifurcated aperture, a feature characteristic for *G.* aff*. rossensis*. Sequenced specimens of this sub-Antarctic species were obtained from South Georgia and southern Patagonia, but its range seems to cover also the Falklands, as specimens showing a bifurcated aperture with a characteristic lip [Fig. 3(1–3)] were also noted in this archipelago. Adult individuals of *G.* aff. *rossensis* and *G. biora* are clearly different from *G.* aff. *subglobosa*, which possesses a single aperture oriented nearly perpendicular to the last suture [Fig. 3(6)]. However, juveniles of all three species can be difficult to distinguish.

In *Cassidulinoides*, molecular delimitation analyses also suggest the presence of three MOTUs/species (Fig. 2); *C. parkerianus* s.s. limited to Patagonia, *C. parvus* to Antarctica, and *C.* aff. *parkerianus* ranging through the Falklands, South Georgia, and South Shetlands in maritime West Antarctica. Except in *C. parkerianus* s.s, they show significant morphological polymorphism. There is a clear morphological distinction between populations of *C. parvus* sampled from South Shetlands and those from the Ross Sea (Fig. 3), although it is not known yet whether their ranges overlap or their morphologies grade into each other (Fig. 1). Individuals from South Shetlands are smaller and more strongly elongated and their adult chambers are less globular [Fig. 3(14)]. Pores are located on tiny projections, causing the test wall to appear hispid. Specimens from the Ross Sea have thick, smooth, finely perforated test walls and relatively few, large chambers. Only the last few chambers uncoil [Fig. 3(15)].

Most specimens of *C.* aff. *parkerianus*, including those from maritime Antarctica, seem to be indistinguishable from the Patagonian *C. parkerianus* s.s. [Fig. 3(7)]; i.e. the chambers are subglobular and after uncoiling they do not increase in size, the test wall is thick, with densely spaced large pores [Fig. 3(9–12)]. However, a second morphotype occurs among specimens from South Georgia. Adult specimens are about twice as long, their chambers increase regularly in size, test walls are thick and smooth, with minute, sparsely-located pores [Fig. 3(8 and 13)]. The chambers are also more inflated towards the aperture compared to most specimens of the first type.

### Genetic variability

**Figure 4:**
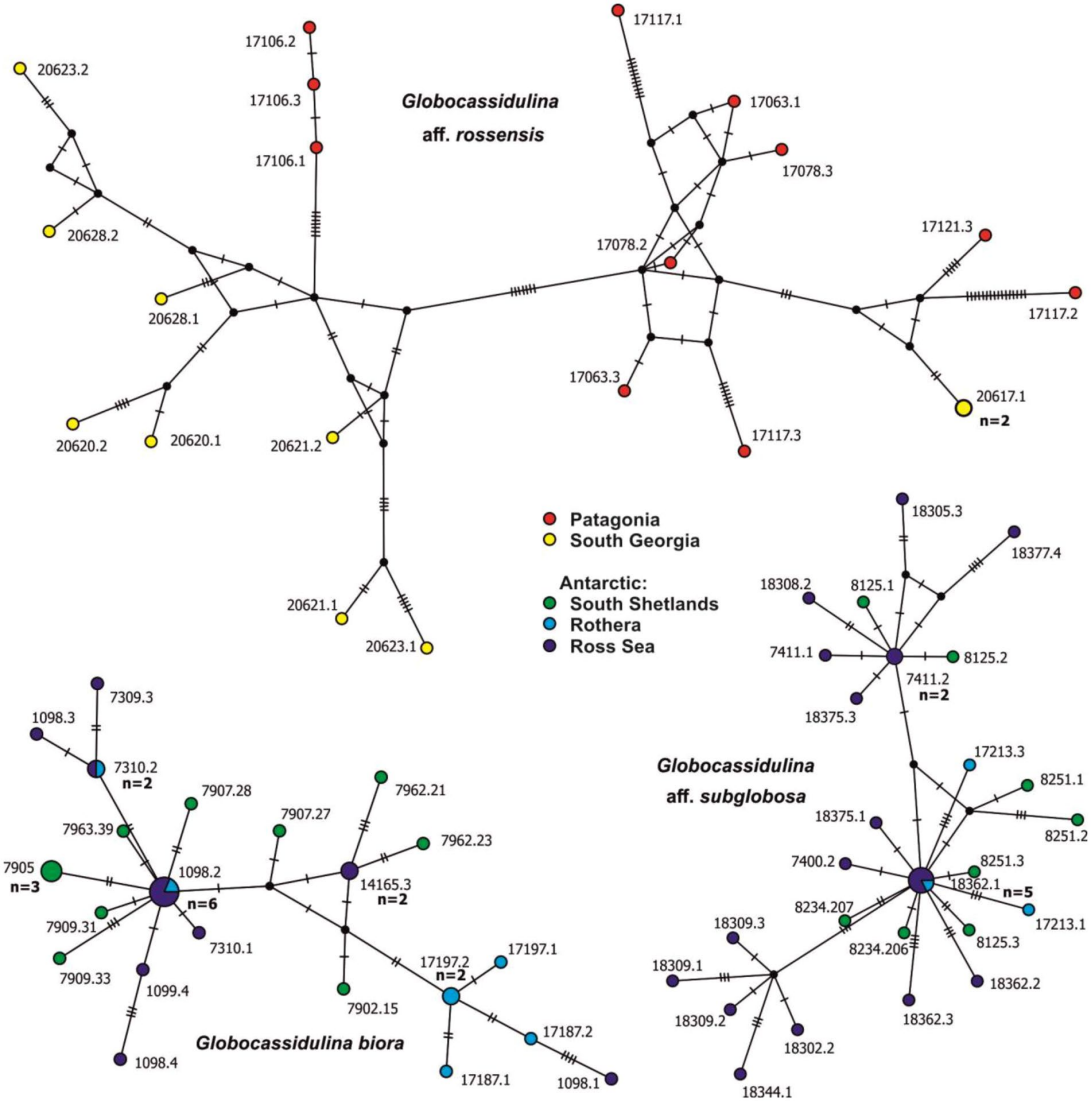
Haplotype networks of different species of Southern Ocean *Globocassidulina* based on matrices constructed from partial SSU rDNA sequences. The area of the circles is proportional to haplotype frequency. Different colors represent different locations.

Genetic dissimilarity in *Globocassidulina* reached 11.2%. *Globocassidulina biora* and *G.* aff. *subglobosa* showed moderate intra-specific genetic dissimilarity of up to 1.8% and 2.3%, respectively (Table 1), while the inter-specific genetic distance between these two Antarctic species reached ~ 4% (Appendix 5). Intra-genomic polymorphism, i.e. the genetic distance between cloned sequences from a single isolate, amounted to 1.3% and 1.8%. This polymorphism was within the diversity range of the two species and there were no indications of inter-species gene exchange. Haplotype network diagrams for *G. biora* and *G. subglobosa* are similar. Both include more than one network center with star-like patterns and sequences from different regions mixed within most sub-groupings (Fig. 4). Haplotypes from the Ross Sea tend to be present in all sub-groups, while haplotypes from South Shetlands are localized in those that are more central. In contrast, the sub-Antarctic species *G.* aff. *rossensis* shows extremely large intra-specific genetic dissimilarity (up to 10.8%, Table 1), greatly surpassing the variability observed within most Antarctic benthic foraminifera currently analyzed^56,57^. It also displays high intra-genomic polymorphism, reaching 7.2% in isolate 17,117. The topology of the haplotype network of this species is strongly reticulate, with long branches and no mixing of sequences from Patagonia and South Georgia (Fig. 4). All haplotypes, except one, were detected in a single isolate only.

**Figure 5:**
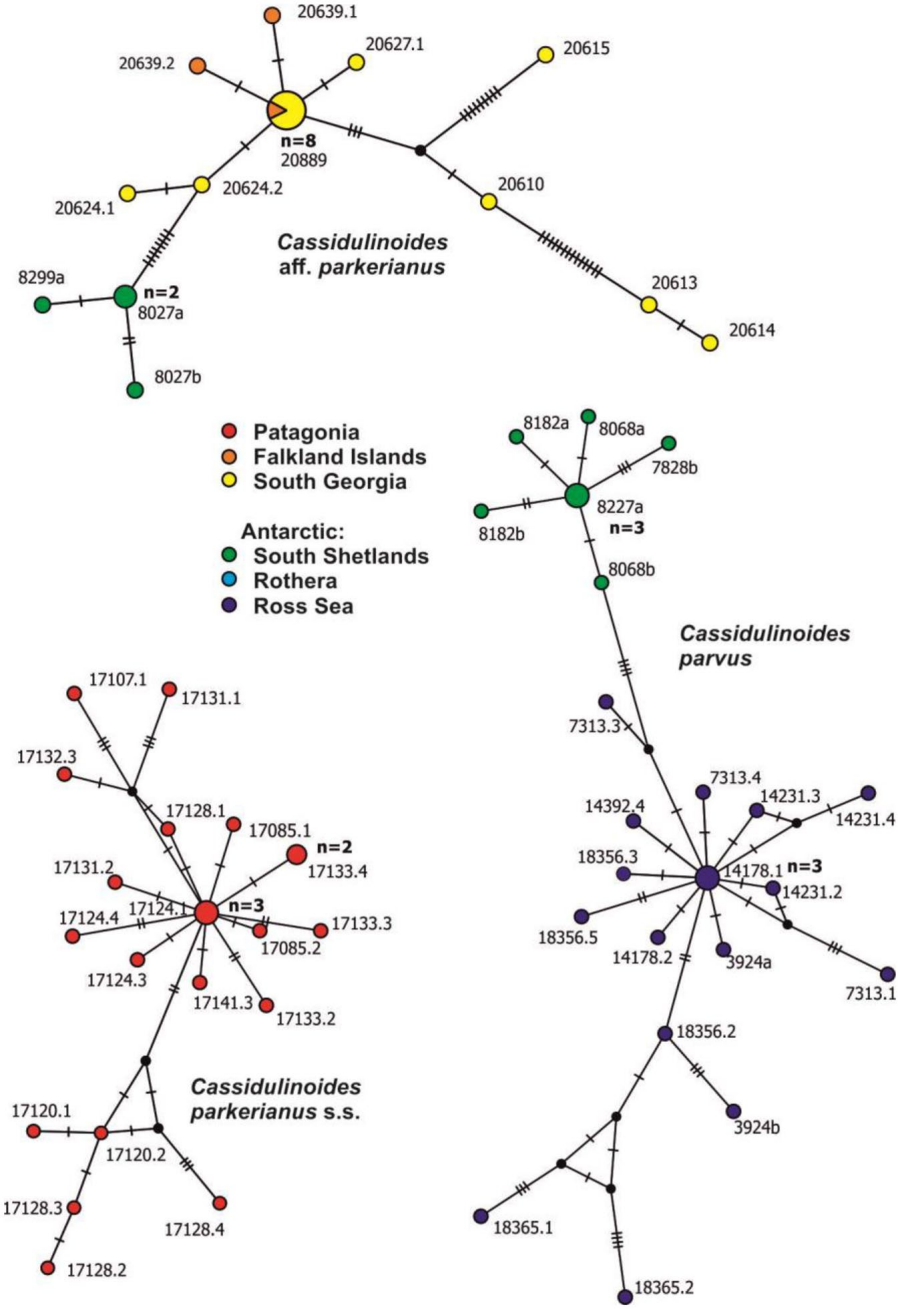
Haplotype networks of different species of *Cassidulinoides*, based on matrices constructed from partial SSU rDNA sequences. The area of the circles is proportional to haplotype frequency. Different colors represent different locations.

Genetic dissimilarity in *Cassidulinoides* ranges up to 9.6% (Table 1). The Patagonian *C. parkerianus* s.s. shows the lowest genetic diversity (up to 1.9%) with a similar level of intra-genomic diversity (up to 1.6%). Its haplotype network displays a large central star-like structure with two prominent branches. Its complexity is similar to that of Antarctic *Globocassidulina* species, but as in *C. parvus*, there is no geographic mixing (Fig. 5). In *C. parvus*, intraspecific genetic dissimilarity is significantly greater, reaching up to 3.9%, while intra-genomic diversity amounts to 0.7%. There is a clear genetic distinction between regional populations, represented by two separate clusters of haplotypes, one grouping sequences from South Shetlands and the other, more developed, grouping sequences from the Ross Sea (Fig. 5). These two groupings are separated by four mutations and genetic dissimilarity of ~ 3% (Appendix 5). In *C.* aff. *parkerianus*, genetic variability reached 3.8% (Table 1), while intra-genomic diversity was very low (0.3%), and was absent in most isolates. The haplotype network of this species, spanning Falkland Islands, South Georgia and South Shetlands, is less resolved and shows longer branches (Fig. 5). Sequences from the Falklands and South Georgia, as well as from two distinct sub-Antarctic morphotypes, are mixed together in the central star-like structure. Antarctic sequences are separated from sub-Antarctic haplotypes by eight mutations and genetic dissimilarity of ~ 1% or more (Appendix 5).

### Demography

**Figure 6:**
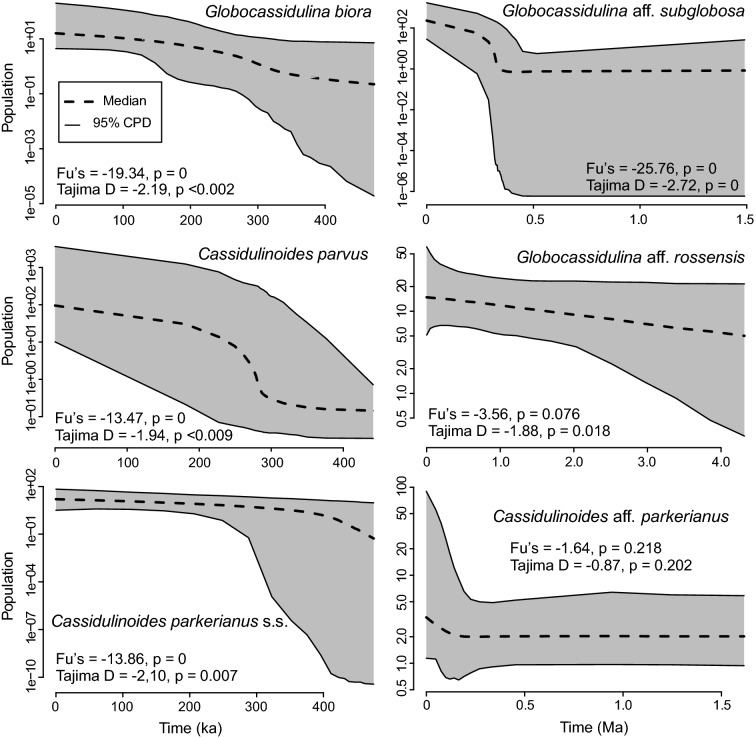
Historical demographic trends of population size constructed using Bayesian skyline plot approach based on SSU rDNA of Antarctic and sub-Antarctic Cassidulinidae. The y-axis (population size) is on a log scale;the x-axis is the time in 10^3^ (left column) and 10^6^ (right column) years before present. Results of neutrality Tajima's D and Fu's Fs tests are indicated in Appendix 6.

Neutrality tests for all three Antarctic species (*G. biora*, *G.* aff. *subglobosa* and *C. parvus*), as well as Patagonian *C. parkerianus* s.s., are statistically significant (Appendix 6 and Fig. 6) and suggest that all the populations are in expansion after possible recent bottlenecks. These results are supported by the eBSP plots (Fig. 6), showing populations size increments in all the cases. Noticeable population growth is observed in the case of *C. parvus* and *G.* aff. *subglobosa,* dating from ~ 300 thousand years ago (kyr) and ~ 400 kyr, respectively. Population growth in *G. biora* and *C. parkerianus* s.s. appears to be more stable, with slight increments occurring, again at ~ 300 kyr and > 400 kyr, respectively. Neutrality tests show modest support for population expansion in *G.* aff. *rossensis* but no statistically significance growth for *C.* aff. *parkerianus*. The eBSP plots reveal only minor population changes in *G.* aff. *rossensis* and *C.* aff*. parkerianus*.

### Time calibrated phylogeny

**Figure 7:**
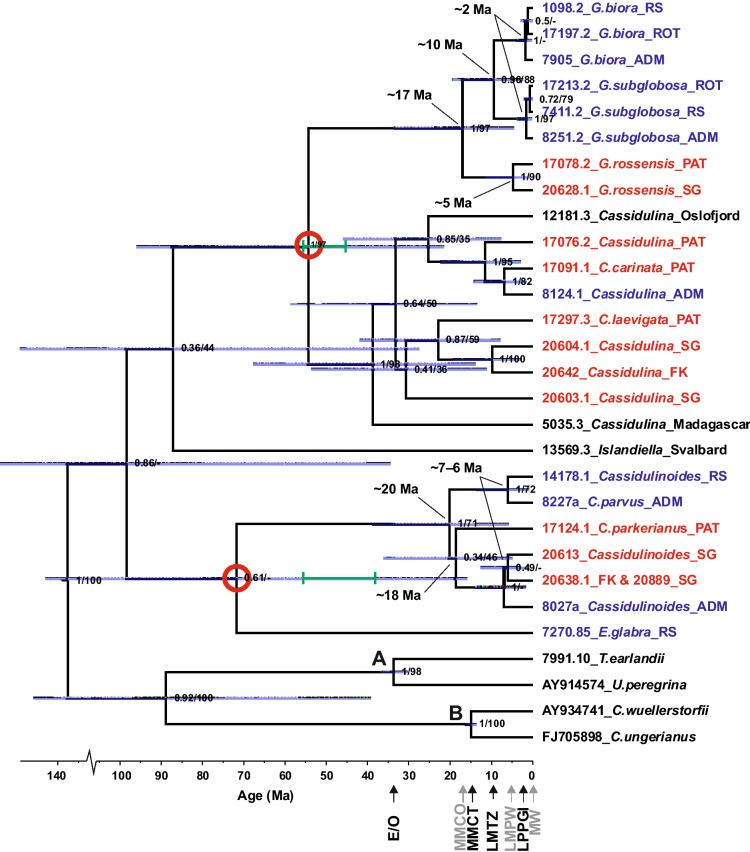
Bayesian time calibrated phylogeny reconstruction using foraminiferal SSU rDNA, showing major divergences in Southern Ocean Cassidulinindae. The posterior probabilities provided above the nodes and supplemented with ML bootstrap values. Sequence ids from Antarctica (*RS* Ross Sea, *ROT* Rothera, Margarite Bay, *ADM* Admiralty Bay, South Shetlands) are marked in blue, from southern Patagonia (*PAT*), Falkland Islands (*FK*) and South Georgia (*SG*) in green, and other in black. Id of sequences used to calibrate the molecular clock are in black; A and B—calibration points. Red circles show divergence events used for testing of the calibration; green bars show age-ranges for these events suggested by the fossil record. Gray bars on nodes show 95% Highest Posterior Density intervals for discussed divergence events; − not recovered in ML phylogenetic reconstruction shown in Appendix 7. Paleoceanographic events marked on the time scale (warm events in grey): *E/O* the Eocene/Oligocene cooling, *MMCO* the mid-Miocene Climatic Optimum, *MMCT* the Middle Miocene Climate Transition, *LMTZ* the Late Miocene strengthening of thermal bathymetric zonation, *LMPW* the Late Miocene to Pliocene warming, *LPPGI* the Late Pliocene to Pleistocene glacial intensification, *MW* the modern warming.

Phylogenetic analyses show that all genera of the Cassidulinidae form well supported clades (Fig. 7). The analyses include a few species from other regions (e.g. *Islandiella* from Svalbard) but their phylogenetic position is generally congruent with morpho-taxonomy. The tree is rooted with closely related rotaliids of family Uvigerinidae and Cibicididae.

According to our time-calibration, the divergence between *Cassidulina* and *Globocassidulina* is strongly supported and dated at ~ 54 Ma, within the range suggested by the fossil record. Thus, the age estimates of evolutionary events in these two genera seem robust. The divergence between *Cassidulinoides* and *Ehrenbergina* has weak support. It also fits within the error bar for the time scale, but at ~ 71 Ma it is 15 million years before the earliest date (56 Ma) based on the fossil record (Fig. 7).

Within the genus *Globocassidulina*, the divergence between Antarctic and sub-Antarctic species took place ~ 17 Ma (Fig. 7). Further radiation of Antarctic *Globocassidulina* with the separation of *G. biora* and *G.* aff. *subglobosa* is dated to ~ 10 Ma. The last diversification of Antarctic *Globocassidulina* started at ~ 2 Ma, while the radiation of sub-Antarctic *G.* aff. *rossensis* probably took place somewhat earlier, ~ 5 Ma.

Divergence within *Cassidulinoides* seems to have started ~ 20 Ma with the separation of Antarctic and sub-Antarctic species. It was followed ~ 2 million years later by the separation of *C. parkerianus* from Patagonia and other regions. A previous divergence within the Antarctic lineages of *C. parvus* and *C.* aff*. parkerianus* occurred ~ 7–6 Ma.

## Discussion

By combining morphological and molecular data on Antarctic and sub-Antarctic Cassidulinidae, our study clarified the taxonomy of this family. We distinguished six species in the genera *Globocassidulina* and *Cassidulinoides*, of which three (*G.* aff. *subglobosa*, *G*. aff. *rossensis* and *C*. aff. *parkerianus*) are possibly new to science. We also established their geographic ranges and obtained data on their genetic variations. The comparison of these data to fossil records and to the geological and climatic history of Antarctic and sub-Antarctic regions allows us to identify four major events that contributed to the evolution of coastal benthic fauna in this area. These events include: (1) isolation of Antarctic fauna during the Early to Middle Miocene climate reorganization; (2) colonization of the Antarctic shelf in the Late Miocene; (3) migration across the Drake Passage during the Late Miocene and Early Pliocene warming periods; (4) radiation of Antarctic species driven by intensified Late Pliocene to Pleistocene glacial cycles. These events are highlighted in Fig. 7 and their detailed consequences are discussed below.

### Separation of Antarctic and sub-Antarctic fauna during early to middle Miocene climate oscillations

Both *Cassidulinoides* and *Globocassidulina* share the timing of separation between their Antarctic and sub-Antarctic species that took place during Early to Middle Miocene (Fig. 7), a long time after the Eocene/Oligocene boundary that marked an important step in the separation of ecosystems on the two sides of the Drake Passage^8^. The divergence between Antarctic *C. parvus* and the predominantly sub-Antarctic *C. parkerianus* s.l. was dated at ~ 20 Ma, while the divergence between Antarctic and sub-Antarctic *Globocassidulina* took place ~ 17 Ma. This was during the onset of the mid-Miocene Climatic Optimum (MMCO) that directly preceded the sharp cooling of the Middle Miocene Climate Transition (MMCT), beginning at ~ 14 Ma. These two Early to Middle Miocene climate shifts facilitated the mid-Miocene reorganization of Earth’s climate system^88^.

The MMCT was marked by a major expansion of the Antarctic ice sheet and global cooling^9^. It was associated with the strengthening of the ACC^89^, the opening of the Drake Passage for deep-water circulation^10^, and associated with strong environmental changes in Antarctica, including cooling^90^, increased seasonality^91^ and salinity fluctuations^92^. These factors increased environmental gradients and the biogeographic isolation of Antarctic fauna and facilitated allopatric speciation, resulting in a series of diversification events in the Southern Ocean^93–97^ and in the deep sea^98^.

Our study confirms the role that the MMCO and MMCT played in the evolution of both genera. However, if we assume that the molecular timing is imperfect, then there are two scenarios that could explain the observed divergences. The first scenario would suggest that the split between Antarctic and sub-Antarctic lineages of *Globocassidulina* and *Cassidulinoides* was a consequence of the increased environmental gradients and strengthening of the biogeographical barrier of the ACC and the PF in the Drake Passage during the MMCT, resulting in vicariant speciation. A second scenario would associate the early divergences of both genera with the MMCO. This warm period was clearly evident in the Southern Ocean^88^, where it lasted from 17 to 15 Ma^9^, but in Antarctica it began ~ 20 Ma^99^. Thus, its timing fits well with the early radiation events in *Cassidulinoides* and *Globocassidulina*. The MMCO was certainly associated with weakening of environmental gradients, which could enable breaching of the Drake Passage in a similar way to that during the later Late Miocene to Pliocene warmth discussed in a later section. This would result in a colonization event, rather than in a split of evolutionary lineages as in the first scenario.

The existing fossil record, although fragmentary, may provide some support for both possibilities. Forms similar to *G. subglobosa*, that could be interpreted as the common ancestor for the three modern species of Southern Ocean *Globocassidulina*, were present continuously since the Late Eocene in Antarctica^41,45^ and southern Patagonia^100,101^. Thus, the split into Antarctic and sub-Antarctic lineages following the MMCT seems plausible. Representatives of *Cassidulinoides* were present since the Early Oligocene in Antarctica^41,47,102^ and Patagonia^101^. However, forms similar to *C. parkerianus* have been reported in Patagonia only since the Middle Miocene^103^, whereas in Antarctica they were already present in the Early Oligocene. This pattern could suggest a mid-Miocene migration of *C. parkerianus* from Antarctica to Patagonia, although at present this scenario remains purely hypothetical and is impossible to test genetically.

### Radiation of Antarctic species during late Miocene bathymetric zonation

Another factor that may have promoted the radiation of Antarctic foraminifera is related to bathymetric zonation. This is best illustrated by the two species of *Globocassidulina* that are found at our Antarctic sites but not at our sub-Antarctic sites. One of them, *G. biora,* seems to be endemic and restricted to the Antarctic shelf where it dominates benthic foraminiferal assemblages at relatively shallow depths, down to ~ 200 m^104,105^. The other species, *G.* aff. *subglobosa*, is morphologically similar to a global, deep-water species^106^ and is considered to have a deeper bathymetric range^27^. Sequences from two specimens of the *G. subglobosa* type from the abyssal Pacific (unpublished) are very different from the Antarctic population of *G.* aff. *subglobosa*, and we cannot exclude the possibility that another genetically distinct species is present in the deep Southern Ocean. Nevertheless, it seems clear that both *G. biora* and *G*. aff. *subglobosa* differ in their bathymetric ranges. This is confirmed by our sampling data. The vast majority of *G. biora* specimens were collected shallower than ~ 100 m, whereas the majority of *G.* aff. *subglobosa* specimens came from depths of ~ 600 m in the Ross Sea (Appendix 1).

Divergence of these two species seems to have occurred in the Late Miocene, probably ~ 10 Ma (Fig. 7). This was a period when radiations among Southern Ocean benthic taxa were being driven by an enhanced thermal bathymetric zonation in the ocean^20^, due to an intensified production of cold and dense Southern Component Water^107^, as well as a time of expanding and over-deepening of the Antarctic continental shelf^108^ after the MMCT. The radiation of *Globocassidulina* could therefore be an example of dispersal from Antarctica to the deep sea through “submergence” following thermohaline circulation, as also suggested for octopuses^98,109^, gastropods^94^ and amphipods^110^. The scenario has to be confirmed by molecular data for abyssal *G. subglobosa* from the Southern Ocean.

The long fossil record of *G. subglobosa* in Antarctica^41,45^ and southern Patagonia ^100,101^ suggests that this species has been present in the area for a long time. Many pre-Miocene records for *G. subglobosa* came from paleoenvironments at water depths of 150–300 m^40^ or significantly shallower than 100 m^45,47,111^, supporting its long-term presence in shallow-water environments. Although it is difficult to compare fossil and modern species, it seems possible that the Late Miocene bathymetric structuring of water column, in combination with broadening and deepening of the Antarctic continental shelf, increased diversity of niches available for colonization, which was an important factor driving the divergence of Antarctic *Globocassidulina* species.

### Breaching biogeographical barriers during the late Miocene to Pliocene warmth

Our results suggest that some Cassidulinidae species could have crossed the Polar Front (PF) and ACC barriers as their molecular and morphologic diversity does not fit a simple pattern of evolution of Antarctic and sub-Antarctic species in isolation since the full opening of the Drake Passage in the Middle Miocene. This applies particularly to *C.* aff. *parkerianus*, the distribution of which spans the Falklands, South Georgia and South Shetlands. Another possible example is *C. parvus.* Although detected so far only in Antarctica, the South Shetlands population of this species differs morphologically and genetically from that in the Ross Sea. The haplotype networks of both populations are separated by just four mutations (Fig. 5) but also include an indel of 23 bases.

This pattern could be interpreted as a result of inter-species hybridization, as has been shown for *Elphidium macellum* from Chilean fiords^112^. Therefore, we suggest that the South Shetlands population of *C. parvus* is a result of hybridization between the Antarctic population from the Ross Sea and an unknown genotype that migrated across the Drake Passage, possibly ~ 6 Ma (Fig. 7) during the Late Miocene. Hybridization may play an important role in facilitating evolutionary change, offering an adaptive advantage to invasive species^113^. This has also been suggested for other Southern Ocean benthic organisms, including the sea spider *Colossendeis megalonyx*^114^ and the ascidian *Cnemidocarpa verrucosa*^115^. A hypothetical sub-Antarctic origin of the morphotype of *C. parvus* in the South Shetlands is supported by its similarity to the Patagonian *C. parvus* of Arellano et al*.*^33^.

If the hybridization hypothesis is accepted, then we would further propose that the South Shetlands, being geographically and ecologically closer to sub-Antarctica than the rest of the Antarctic, could act as a place where sub-Antarctic and Antarctic populations may occasionally mix. The possibility of breaching the biogeographical barrier of the ACC and the PF in the current warming climate has been highlighted recently^116,117^. For benthic foraminifera, such migration would be mediated most easily by water-borne propagules^118,119^, circumventing the need for rafting. In fact, modern gene flow between southern Patagonia and South Shetlands has already been suggested for the rotaliid foraminifera *Trifarina earlandi* and *Pullenia subcarinata*^57^. A similar process could have occurred during the Late Miocene to Pliocene warmth^9^, which is viewed as an analog for present climate changes^120^. If so, it possibly included the migration of *G.* aff. *rossensis* from Patagonia to the Falklands and South Georgia. However, the establishment of populations on South Georgia might also have coincided with the emergence of this island ~ 10–7 Ma^121^. Further to the south, it appears that either the PF, or a number of ocean eddies^122,123^, may have reached the Antarctic Peninsula on at least three occasions during the Late Miocene to Pliocene^124^. If true, trans-PF migrations could have facilitated open gene flow across the Drake Passage in some foraminifera and other marine invertebrates^125,126^.

### Radiation and demographic changes driven by intensified glacial cycles

Late Pliocene to Pleistocene was a time when glacial cycles intensified^127,128^, leading to a repeated fragmentation of populations in the Antarctic marine realm followed by allopatry and recolonization of the continental shelf during interglacials^15,129^. This proposed process has been termed a “diversity pump”^17,18^ and is believed to drive intensified radiation in many Southern Ocean organisms^96,97,130–132^, including planktonic foraminifera^133^.

The last diversification of Antarctic *Globocassidulina* starting ~ 2 Ma (Fig. 7), only slightly post-dating the earliest fossil record of *G. biora,* dated at ~ 3 Ma^44,49,134^, may be a consequence of this intensified glacial activity. The star-like topologies of haplotype networks in most species of the Cassidulinidae (Figs. 4 and 5), and other Antarctic benthic foraminifera^57^, may reflect the same phenomenon. In *G. biora* and *G. subglobosa,* there appear to have been multiple diversification centers (Fig. 4) and sequences from different regions are mixed within most sub-groupings, suggesting active gene flow. These networks are clearly different from single star-like topologies that reflect a single refugium, bottleneck and subsequent population expansion^127,135^. Such complex topologies hint at an intricate dispersion history during the last glaciations, involving (1) the survival of small populations in multiple glacial refugia, or alternately, (2) survival of larger populations in deeper water^13,113,129,136–138^, and re-colonization of the Antarctic continental shelf during subsequent interglacials.

In the case of *G.* aff. *subglobosa*^27,139,140^, the second hypothesis might be applicable, although demographic data indicating population expansion at ~ 400 kyr (Fig. 6) suggest a rather strong impact of glaciation on its population size. For *G. biora*, restricted to the continental shelf of Antarctica, the refugia scenario seems more likely. In fact, *G. biora* is renowned as a glacier proximal species^35,36,105,141,142^. A spinose variant of *G. biora*, described from near grounding line or sub-ice shelf environments in the Ross Sea^143^, could be an example of a highly specialized ecotype, especially well adapted to near-glacial conditions. Thus, the representatives of this Antarctic species are potentially well predisposed to survive former glaciations in glacial refugia near the edges of the continental shelf^12^.

The population structure of sub-Antarctic *G.* aff. *rossensis* is more heterogeneous than in its Antarctic counterparts. It shows a complex, reticulate haplotype network topology, excluding a bottle neck, and no signs of recent genetic mixing between populations from Patagonia and South Georgia (Fig. 4). Moreover, it seems that the last glaciations had only a limited impact on its population size (Fig. 6).

The population structure of *Cassidulinoides* species is different. Although the haplotype networks of the Patagonian *C. parkerianus* s.s. and *C. parvus* from the Ross Sea resemble those of Antarctic *Globocassidulina*, there is no geographic mixing, suggesting restricted gene flow (Fig. 5). The population structures of these two species are similar and consist of prominent star-like structures, reflecting a bottleneck and subsequent population expansion, as confirmed by neutrality tests (Fig. 6 and Appendix 6). The Antarctic species (*G. parvus*) must have been affected by a drastic decrease in living space during the last glaciations^130^. However, the Patagonian population of *C. parkerianus* s.s. was less impacted by the last glacial period, which mainly occurred along the Pacific side of South America, leaving the Atlantic coast ice free^132,144^. Perhaps cooling and other environmental changes associated with glacial expansion severely restricted natural habitats of *C. parkerianus* s.s., as postulated for *Galaxias maculatus*, a fish species inhabiting Chilean fiords south from 42°S^145^.

According to the eBSP reconstructions for the three Antarctic species (*G. biora*, *G.* aff. *subglobosa*, and *C. parvus*), their demographic expansion took place between roughly 500 and 200 kyr (Fig. 6), which may suggest a stronger reduction of their populations during older glacial cycles than during the last one, either due to the larger extent of the penultimate glaciations or to an increasing adaptation of Antarctic biota to extreme glacial conditions. As discussed earlier for *G. biora*^143^, and supported by its stable population growth (Fig. 6), increasing adaptation of this species to glacier-proximal conditions during past glacial maxima seems more likely.

## Concluding remarks

Our combined morphological and taxonomic study has clarified the taxonomy and biogeography of Antarctic and sub-Antarctic members of the Cassidulinidae. It has also demonstrated how the complex interplay between environmental changes driven by the tectonics, climate and oceanography that have characterized the Scotia Sea and the Antarctic Peninsula throughout the Cenozoic, and particularly since the Early Miocene (~ 20 Ma), have profoundly influence the evolution and biogeography of this important foraminiferal family. In addition to being a major component of marine benthic communities, foraminifera have an excellent fossil record, making them particularly well suited to studies of this kind. It is therefore reassuring that our results mirror those obtained using similar approaches focused on metazoan organisms as varied as fish^93^, octopuses^98,109^, shrimps^137^ and limpets^96,126^, as well as macroalgae^146^, all of which have poor fossil records. The utility of foraminifera as proxies for macroecological changes over historical and geological time scales has been demonstrated in a number of previous studies^147,148^.

The biogeographic barrier created by the PF and ACC^25^ has impeded faunal dispersal during past interglacials and warm periods^17^. Currently, Antarctica is experiencing some of the fastest climate change in its history^149^, probably faster than at any time during the Cenozoic. This is likely to have major ecological consequences. With ongoing warming and the southward shift of marine currents, areas to the south of the PF and ACC are increasingly exposed to species invasion^116,117,150^. Our data suggest that the Late Miocene to Pliocene warming could have been an analogous period during which biogeographic barriers across the Drake Passage were breached. The environmental consequences of modern warming will create further challenges likely to disturb and disrupt biological communities. These include those arising from acidification, increased carbonate dissolution, decreased salinity resulting from freshwater runoff, decreased bottom-water oxygenation, and increases in sedimentation and iceberg scouring^150,151^. Studies with a geological perspective, such as ours, can provide a broader context for how these complex environmental changes, combined with increased connectivity across the Drake Passage, might refashion communities and biogeographic patterns in this critical region.

## Supplementary Information

Supplementary Information 1.

Supplementary Information 2.

Supplementary Information 3.

Supplementary Information 4.

Supplementary Information 5.

Supplementary Information 6.

Supplementary Information 7.
