# Supplementary material for: Cenozoic climatic changes drive evolution and dispersal of coastal benthic foraminifera in the Southern Ocean
Source: Sci Rep. 2021 Oct 6;11:19869. doi: 10.1038/s41598-021-99155-6 (PMC8494791; doi:10.1038/s41598-021-99155-6)
Supplement: Supplementary file 1 — Supplementary Information 1. [file 41598_2021_99155_MOESM1_ESM.docx]

**Appendix 1.** Accession numbers and sample locations. McM = McMurdo

| **Taxon** | **ID** | **GeneBank accession #** | **Location** | **Latitude** | **Longitude** | **Water depth (m)** | **No. of spec.** |
| --- | --- | --- | --- | --- | --- | --- | --- |
| *Globocassidulina biora* | 1098 | LN873581, LN873583, LN873587, LN873588 | New Harbor, McM Sound | 77°34.318' | 163°30.368' | 15 | 1 |
|  | 1099 | LN873582, LN873586 | New Harbor, McM Sound | 77°34.318' | 163°30.368' | 15 | 1 |
|  | 7309 | LN873589, LN873591 | Double Curtain, McM Sound | 77°39.622' | 163°32.184' | 17 | 1 |
|  | 7310 | LN873592, LN873593 | Double Curtain, McM Sound | 77°39.622' | 163°32.184' | 17 | 3 |
|  | 7902 | LN873574 | Admiralty Bay, KG7 | 62°06.220' | 58°19.676' | 30 | 1 |
|  | 7905 | LN873570 | Admiralty Bay, KG7 | 62°06.220' | 58°19.676' | 30 | 1 |
|  | 7907 | LN873573, LN873575 | Admiralty Bay, KG7 | 62°06.220' | 58°19.676' | 30 | 1 |
|  | 7909 | LN873576, LN873577 | Admiralty Bay, KG7 | 62°06.220' | 58°19.676' | 30 | 1 |
|  | 7931 | LN873571 | Admiralty Bay, KG12 | 62°09.624' | 58°30.157' | 116 | 1 |
|  | 7962 | LN873578, LN873579 | Admiralty Bay, KG5 | 62°06.247' | 58°19.777' | 35 | 1 |
|  | 7963 | LN873580 | Admiralty Bay, KG5 | 62°06.247' | 58°19.777' | 35 | 1 |
|  | 7964 | LN873572 | Admiralty Bay, KG5 | 62°06.247' | 58°19.777' | 35 | 1 |
|  | 14165 | LN873604, LN873605 | McMurdo Jetty, McM Sound | 77°51.714' | 166°65.903' | 21-30 | 1 |
|  | 14194 | LN873606, LN873607 | McMurdo Jetty, McM Sound | 77°51.714' | 166°65.903' | 21-30 | 1 |
|  | 17187 | LN873597 to LN873599 | Rothera, site 40 | 67°34.16' | 68° 07.88' | 8 | 1 |
|  | 17197 | LN873595, LN873596 | Rothera, site 206 | 67°34.11' | 68°12.29' | 515 | 1 |
|  | 17225 | LN873601, LN873602 | Rothera, site 206 | 67°34.11' | 68°12.29' | 515 | 1 |
| *Globocassidulina* aff. *subglobosa* | 7400 | MZ367491, MZ367492 | Double Curtain, McM Sound | 77°39,622' | 163°32.184' | 17 | 4 |
|  | 7411 | MZ367427, MZ367489, MZ367490 | New Harbour, McM Sound | 77°34.576' | 163°31.702 | 28 | 8 |
|  | 8125 | MZ367435, MZ367496, MZ367495 | Admiralty Bay, KG13a | 62°09.461' | 58°29.737' | 108 | 1 |
|  | 8234 | MZ367436, MZ367437 | Admiralty Bay, KG20 | 62°09.053' | 58°30.435' | 249 | 2 |
|  | 8251 | MZ367434, MZ375503, MZ375504 | Admiralty Bay, KG19 | S side of Napier Rock | | 40 | 3 |
|  | 17213 | MZ367438, MZ367439, MZ367440 | Rothera, site 186 | 67°35.35' | 68°11.80' | 230 | 1 |
|  | 18299 | MZ367432 | Ross Sea NBP15-2A, G1 | 76° 44.199' | 165° 33.447' | 630 | 1 |
|  | 18300 | MZ367428 | Ross Sea NBP15-2A, G1 | 76° 44.199' | 165° 33.447' | 630 | 1 |
|  | 18302 | MZ367429 | Ross Sea NBP15-2A, G1 | 76° 44.199' | 165° 33.447' | 630 | 1 |
|  | 18305 | MZ367431 | Ross Sea NBP15-2A, G1 | 76° 44.199' | 165° 33.447' | 630 | 1 |
|  | 18308 | MZ367430 | Ross Sea NBP15-2A, G2 | 76° 44.280' | 165° 32.880' | 639 | 1 |
|  | 18309 | MZ367494, MZ367433, MZ367493 | Ross Sea NBP15-2A, G2 | 76° 44.280' | 165° 32.880' | 639 | 1 |
|  | 18344 | MZ367423 | Ross Sea NBP15-2A, G1 | 76° 44.199' | 165° 33.447' | 630 | 1 |
|  | 18362 | MZ367424, MZ367485, MZ367486 | Ross Sea NBP15-2A, G1 | 76° 03,453' | 170° 23.381' | 600 | 1 |
|  | 18375 | MZ367425, MZ367487, MZ367488 | Ross Sea NBP15-2B, BC1 | 76° 42.377' | 179° 07.149' | 174 | 1 |
|  | 18377 | MZ367426 | Ross Sea NBP15-2B, BC1 | 76° 42.377' | 179° 07.149' | 174 | 1 |
| *Globocassidulina* aff. *C. rossensis* | 17063 | MZ367442, MZ367443 | Beagle Channel, BF11B | 54°53.28' | 69°38.10' | 18 | 1 |
|  | 17078 | MG980213, MG980214 | Beagle Channel, BF11B | 54°53.28' | 69°38.10' | 18 | 1 |
|  | 17106 | MG980208 to MG980210 | Beagle Channel, BF11B | 54°53.28' | 69°38.10' | 18 | 1 |
|  | 17117 | MG980211, MZ367499, MZ367421 | Beagle Channel, BF11B | 54°53.28' | 69°38.10' | 18 | 1 |
|  | 17121 | MG980212 | Beagle Channel, BF11B | 54°53.28' | 69°38.10' | 18 | 1 |
|  | 20617 | MW834404 | South Georgia, SG16 | 54 21.181' | 36°22.948' | 136 | 1 |
|  | 20618 | MW834403 | South Georgia, SG16 | 54 21.181' | 36°22.948' | 136 | 1 |
|  | 20620 | MW834407, MW834408 | South Georgia, SG3 | 54°04.250' | 36°56.812' | 250 | 1 |
|  | 20621 | MW834402, MW834406 | South Georgia, SG3 | 54°04.250' | 36°56.812' | 250 | 1 |
|  | 20623 | MW834405, MW834410 | South Georgia, SG9 | 54°12.044' | 36°34.253' | 60 | 1 |
|  | 20628 | MW834409, MW834411 | South Georgia, SG17 | 54°09.533' | 36°41.583' | 92 | 1 |
| *Cassidulina* spp. | 7642 | MZ367457 | Beagle Channel, Ushuaia | Bahia Lapataia | | 20 | 1 |
|  | 8124 | MZ367483, MZ367484 | Admiralty Bay, KG13a | 62°09.461' | 58°29.737' | 108 | 1 |
|  | 17076 | MZ367452 to MZ367454 | Beagle Channel, BF7 | 54°48.18' | 69°39.29' | 182 | 1 |
|  | 17082 | MZ367455, MZ367459 | Beagle Channel, BF39 | 54°09.12' | 70°07.55' | 169 | 1 |
|  | 17088 | MZ367460, MZ367461 | Beagle Channel, BF50 | 54°52.217' | 68°146.36' | 16 | 1 |
|  | 17091 | MZ367419, MZ367458 | Beagle Channel, BF19 | 54°55.98' | 69°15.36' | 220 | 1 |
|  | 17108 | MZ367448 to MZ367450 | Beagle Channel, BF14 | 54°56.19' | 69°28.41' | 85 | 1 |
|  | 17112 | MZ367463 | Beagle Channel, BF55 | 55°55.80' | 67°18.14' | 22 | 1 |
|  | 17113 | MZ367420, MZ367451 | Beagle Channel, BF7 | 54°48.18' | 69°39.29' | 182 | 1 |
|  | 17156 | MZ367462 | Beagle Channel, BF50 | 54°52.217' | 68°146.36' | 16 | 1 |
|  | 17157 | MZ367456 | Beagle Channel, BF50 | 54°52.217' | 68°146.36' | 16 | 1 |
|  | 17297 | MZ367417, MZ367446, MZ367447 | Beagle Channel, BF20 | 54°56.06' | 69°12.46' | 185 | 1 |
|  | 20603 | MW834398, MW834399 | South Georgia, SG2 | 54°05.670' | 36°58.223' | 49 | 1 |
|  | 20604 | MW834400 | South Georgia, SG2 | 54°05.670' | 36°58.223' | 49 | 1 |
|  | 20642 | MW834358 | Falkland Is, FK2 | 51°39.665' | 57°42.459' | 40 | 1 |
|  | 20643 | MW834360 | Falkland Is, FK2 | 51°39.665' | 57°42.459' | 40 | 1 |
|  | 20645 | MW834354 | Falkland Is, FK2 | 51°39.665' | 57°42.459' | 40 | 1 |
|  | 20646 | MW834355 | Falkland Is, FK2 | 51°39.665' | 57°42.459' | 40 | 1 |
|  | 20648 | MW834353 | Falkland Is, FK2 | 51°39.665' | 57°42.459' | 40 | 1 |
|  | 20652 | MW834359 | Falkland Is, FK2 | 51°39.665' | 57°42.459' | 40 | 1 |
|  | 20653 | MW834362 | Falkland Is, FK2 | 51°39.665' | 57°42.459' | 40 | 1 |
|  | 20654 | MW834352 | Falkland Is, FK2 | 51°39.665' | 57°42.459' | 40 | 1 |
|  | 20655 | MW834361 | Falkland Is, FK2 | 51°39.665' | 57°42.459' | 40 | 1 |
|  | 20656 | MW834357 | Falkland Is, FK2 | 51°39.665' | 57°42.459' | 40 | 1 |
|  | 20657 | MW834356 | Falkland Is, FK2 | 51°39.665' | 57°42.459' | 40 | 1 |
| *Cassidulinoides parvus* RS | 3924 | MZ367379, MZ367380 | Terra Nova Bay | 74°40.28' | 164°04.11' | 25 | 3 |
|  | 7313 | MZ367381, MZ367382, MZ367383 | Double Curtain | 77°39.622' | 163°32.184' | 17 | 1 |
|  | 14178 | MZ367384, MZ367385 | McMurdo Jetty | 77°51.714' | 166°65.903' | 21-30 | 1 |
|  | 14231 | MZ367386 to MZ367389 | McMurdo Jetty | 77°51.714' | 166°65.903' | 21-30 | 1 |
|  | 14392 | MZ367390, MZ367391 | Cape Bernacchi | 77°31.743' | 163° 47.068’ | 18-30 | 1 |
|  | 18356 | MZ367392 to MZ367394 | Ross Sea NBP15-2A, G4 | 76°15.612' | 166°25.026' | 147 | 1 |
|  | 18365 | MZ367497, MZ367498 | Ross Sea NBP15-2A, BC1 | 76° 42.377' | 179° 07.149' | 174 | 1 |
| *Cassidulinoides parvus* ADM | 7795 | MZ367395 | Admiralty Bay, KG8 | 62°09.650' | 58°34.774' | 119 | 1 |
|  | 7828 | MZ367396, MZ367397 | Admiralty Bay, KG9 | 62°09.623' | 58°34.510' | 115 | 1 |
|  | 8068 | MZ367398, MZ367399 | Admiralty Bay, KG13a | 62°09.461' | 58°29.737' | 108 | 3 |
|  | 8182 | MZ367400, MZ367401 | Admiralty Bay, KG14 | 62°09.290' | 58°29.439' | 100 | 1 |
|  | 8227 | LN873758 | Admiralty Bay, KG20 | 62°09.053' | 58°30.435' | 249 | 1 |
| *Cassidulinoides* aff. *C. parkerianus* | 20889 | MW834368 | South Georgia, SG10 | 54°12.759' | 36°33.677' | 114 | 1 |
|  | 20890 | MW834369 | South Georgia, SG10 | 54°12.759' | 36°33.677' | 114 | 1 |
|  | 20891 | MW834370 | South Georgia, SG10 | 54°12.759' | 36°33.677' | 114 | 1 |
|  | 20893 | MW834371 | South Georgia, SG10 | 54°12.759' | 36°33.677' | 114 | 1 |
|  | 20894 | MW834372 | South Georgia, SG10 | 54°12.759' | 36°33.677' | 114 | 1 |
|  | 20608 | MW834367 | South Georgia, SG27 | 54°09.372' | 36°38.426' | 136 | 1 |
|  | 20610 | MW834363 | South Georgia, SG27 | 54°09.372' | 36°38.426' | 136 | 1 |
|  | 20615 | MW834366 | South Georgia, SG9 | 54°12.044' | 36°34.253' | 60 | 1 |
|  | 20624 | MW834412, MW834413 | South Georgia, SG9 | 54°12.044' | 36°34.253' | 60 | 1 |
|  | 20627 | MW834416, MW834417 | South Georgia, SG17 | 54°09.533' | 36°41.583' | 92 | 1 |
|  | 20638 | MW834418 | Falkland Is, FK2 | 51°39.665' | 57°42.459' | 40 | 1 |
|  | 20639 | MW834414, MW834415 | Falkland Is, FK2 | 51°39.665' | 57°42.459' | 40 | 1 |
|  | 8027 | MZ367402, MZ367403 | Admiralty Bay, K13a | 62°09.461' | 58°29.737' | 108 | 1 |
|  | 8299 | MZ367404, MZ367405 | Admiralty Bay, KG22 | 62°05.610' | 58°22.944' | 233 | 1 |
|  | 20613 | MW834364 | South Georgia, SG9 | 54°12.044' | 36°34.253' | 60 | 1 |
|  | 20614 | MW834365 | South Georgia, SG9 | 54°12.044' | 36°34.253' | 60 | 1 |
| *Cassidulinoides parkerianus* s.s. | 17085 | MZ367472, MZ367473 | Beagle Channel, BF50 | 54°52.217' | 68°146.36' | 16 | 1 |
|  | 17107 | MZ367482 | Beagle Channel, BF50 | 54°52.217' | 68°146.36' | 16 | 1 |
|  | 17120 | MZ367444, MZ367445 | Beagle Channel, BF11B | 54°53.28' | 69°38.10' | 18 | 1 |
|  | 17124 | MZ367469, MZ367474, MZ367467 | Beagle Channel, BF20 | 54°56.06' | 69°12.46' | 185 | 1 |
|  | 17128 | MZ367464 to MZ367466, MZ367479 | Beagle Channel, BF11D | near BF11B | | 8 | 1 |
|  | 17131 | MZ367422, MZ367475, MZ367481 | Beagle Channel, BF7 | 54°48.18' | 69°39.29' | 182 | 1 |
|  | 17132 | MZ367471, MZ367480 | Beagle Channel, BF15 | 54°57.46' | 69°29.28' | 23 | 1 |
|  | 17133 | MZ367468, MZ367476, MZ367478 | Beagle Channel, BF15 | 54°57.46' | 69°29.28' | 23 | 1 |
|  | 17141 | MZ367470, MZ367477 | Beagle Channel, BF50 | 54°52.217' | 68°146.36' | 16 | 1 |
| *Ehrenbergina glabra* | 7270 | MZ367406 to MZ367408 | Ferrar I | 77°36.438' | 163°41.984' | 24 | 1 |
|  | 7290 | MZ367409 to MZ367411 | Ferrar I | 77°36.438' | 163°41.984' | 24 | 1 |
|  | 7541 | MZ367412 to MZ367414 | Cape Armitage | 77°51.310' | 166° 40.070 | 18-30 | 1 |
| *Islandiella helenae* | 13569 | MZ367415 | Svalbard | 76°59.686' | 15°56.008' | 137 | 1 |
| *Cassidulina* sp. | 5035 | MZ367416 | Madagascar | - | - | - | 1 |
| *Cassidulina laevigata* | 12181 | MZ367418 | Norway | Oslofjord | | 100 | 1 |
