# Supplementary material for: Cenozoic climatic changes drive evolution and dispersal of coastal benthic foraminifera in the Southern Ocean
Source: Sci Rep. 2021 Oct 6;11:19869. doi: 10.1038/s41598-021-99155-6 (PMC8494791; doi:10.1038/s41598-021-99155-6)
Supplement: Supplementary file 2 — Supplementary Information 2. [file 41598_2021_99155_MOESM2_ESM.docx]

**Appendix 2.** ABGD results.

**Step 1. Results of the ABGD analysis on the entire dataset.**

Initial Partition with prior maximal distance P=2.78e-03

Distance JC69 Jukes-Cantor MinSlope=1.500000

Download (left click and save) or see below the tree file corresponding to this partition: click here

Group[ 1 ] n: 85 ;id: 18344.1_Globocassidulina_RS 18362.1_Globocassidulina_RS 18362.2_Globocassidulina_RS 18362.3_Globocassidulina_RS 18375.1_Globocassidulina_RS 18375.2_Globocassidulina_RS 18375.3_Globocassidulina_RS 18377.4_Globocassidulina_RS 7411.1_G.subglobosa_NH 7411.2_G.subglobosa_NH 7411.3_G.subglobosa_NH 7400.2_G.subglobosa_NH 7400.3_G.subglobosa_NH 18300.1_G.subglobosa_RS 18302.2_G.subglobosa_RS 18308.2_G.subglobosa_RS 18305.3_G.subglobosa_RS 18299.3_G.subglobosa_RS 18309.3_G.subglobosa_RS 18309.2_G.subglobosa_RS 18309.1_G.subglobosa_RS 8234.206 8234.207 17213.1_Globocassidulina_biora 17213.2_Globocassidulina_biora 17213.3_Globocassidulina_biora 8251.3_G.subglobosa_ADM 8251.2_G.subglobosa_ADM 8251.1_G.subglobosa_ADM 8125.3_Cassidulina_ADM 8125.2_ Globocassidulina _ADM 8125.1_ Globocassidulina_ADM 7905_G.biora_ADM 7931_G.biora_ADM 7964_G.biora_ADM 7907.27_G.biora_ADM 7902.15_G.biora_ADM 7907.28_G.biora_ADM 7909.31_G.biora_ADM 7909.33_G.biora_ADM 7962.21_G.biora_ADM 7962.23_G.biora_ADM 7963.39_G.biora_ADM 1098.4_G.biora_NH 1099.4_G.biora_NH 1098.2_G.biora_NH 1099.1 1098.1 1098.3 7309.1_G.biora_NH 7309.3_G.biora_NH 7310.1_G.biora_NH 7310.2_G.biora_NH 17197.1_G.biora_ROT 17197.2_G.biora_ROT 17187.3_G.biora_ROT 17187.2_G.biora_ROT 17187.1_G.biora_ROT 17225.1_Globocassidulina_biora 17225.2_Globocassidulina_biora 14165.3_Globocassidulina_biora 14165.1_Globocassidulina_biora 14194.2_Globocassidulina_biora 14194.3_Globocassidulina_biora 20618.1_ Globocassidulina_SG 20623.1_ Globocassidulina_SG 20628.2_Globocassidulina_SG 20623.2_ Globocassidulina _SG 20620.1_Globocassidulina_SG 20620.2_Globocassidulina_SG 20621.1_Globocassidulina_SG 20621.2_Globocassidulina_SG 20617.1_ Globocassidulina_SG 20628.1_Globocassidulina_SG 17063.1_G.minuta_BF11B 17063.3_ Globocassidulina_BF11B 17078.2_G.rosensis_BF11B 17078.3_G.rosensis_BF11B 17117.3_G.rosensis_BF11B 17106.3_G.rosensis_BF11B 17106.2_G.rosensis_BF11B 17106.1_G.rosensis_BF11B 17121.3_G.rosensis_BF11B 17117.1_G.rosensis_BF11B 17117.2_G.rosensis_BF11B

Group[ 2 ] n: 37 ;id: 17297.2_C.laevigata_BF20 17297.3_C.laevigata_BF20 17108.1_C.carinata_BF14 17108.2_C.carinata_BF14 17108.3_C.carinata_BF14 20603.1_Cassidulina_SG 20603.2_Cassidulina_SG 20654_Cassidulina_FK 20645_Cassidulina_FK 20646_Cassidulina_FK 20648_Cassidulina_FK 8124.1_Cassidulina_ADM 8124.2_Cassidulina_ADM 17113.1_Cassidulina_BF07/ 17113.2_Cassidulina_BF07/ 17076.1_Cassidulina_BF07/ 17076.2_Cassidulina_BF07/ 17076.3_Cassidulina_BF07/ 17082.1_Cassidulina_BF36 17157.2_Cassidulina_BF50 7642.1_Cassidulina_USH 17297.1_C.laevigata_BF20 17091.1_C.carinata_BF19 17091.3_C.carinata_BF19 17082.2_Cassidulina_BF36 17088.1_Cassidulina_BF50 17088.3_Cassidulina_BF50 17156.3_Cassidulina_BF50 17112_C.crassa_BF55 20642_Cassidulina_FK 20643_Cassidulina_FK 20652_Cassidulina_FK 20655_Cassidulina_FK 20656_Cassidulina_FK 20657_Cassidulina_FK 20604.1_Cassidulina_SG 20653_Cassidulina_FK

Group[ 3 ] n: 64 ;id: 20624.1_Cassidulinoides_SG 20624.2_Cassidulinoides_SG 20627.1_Cassidulinoides_SG 20627.2_Cassidulinoides_SG 20638.1_Cassidulinoides_FK 20639.1_Cassidulinoides_FK 20639.2_ Cassidulinoides _FK 20608_Cassidulinoides_parkeriensis_SG 20610_Cassidulinoides_parkeriensis_SG 20613_Cassidulinoides_parkeriensis_SG 20614_Cassidulinoides_parkeriensis_SG 20615_Cassidulinoides_SG 8068a_C.parkerianus_ADM 8227a_C.parkerianus_ADM 7828a_C.parkerianus_ADM 8182a_C.parkerianus_ADM 8068b_C.parkerianus_ADM 7795a_C.parkerianus_ADM 8182b_C.parkerianus_ADM 7828b_C.parkerianus_ADM 7313.3_Cassidulinoides_McM 14178.1_Cassidulinoides 14392.1_Cassidulinoides 14231.1_Cassidulinoides 7313.4_Cassidulinoides 3924a_C.parkerianus_TB 14178.2_Cassidulinoides 14231.2_Cassidulinoides 18356.3_Cassidulionides_large_RS 14392.4_Cassidulinoides 14231.3_Cassidulinoides 14231.4_Cassidulinoides 18365.2_Cassidulinoides_RS 18365.1_Cassidulinoides_RS 18356.5_Cassidulinoides_large_RS 7313.1_Cassidulinoides_McM 3924b_C.parkerianus_TB_DQ452689 18356.2_Cassidulinoides_large_RS 8027b_C.porrectus_ADM 8027a_C.porrectus_ADM 8299b_C.porrectus_ADM 8299a_C.porrectus_ADM 17120.1_Cassidulinoides_BF11B 17120.2_Cassidulinoides_BF11B 17128.3_C.parkerianus_BF 17128.2_C.parkerianus_BF 17128.4_C.parkerianus_BF 17124.4_C.parkerianus_BF 17133.2_C.parkerianus_BF 17124.1_C.parkerianus_BF 17141.1_C.parkerianus_BF 17132.1_C.parkerianus_BF 17085.2_C.parkerianus_BF 17085.1_C.parkerianus_BF 17124.3_C.parkerianus_BF 17131.2_C.parkerianus_BF 17133.4_C.parkerianus_BF 17141.3_C.parkerianus_BF 17133.3_C.parkerianus_BF 17128.1_C.parkerianus_BF 17132.3_C.parkerianus_BF 17131.1_C.parkerianus_BF 17131.3_C.parkerianus_BF 17107.1_C.parkerianus_BF

Group[ 4 ] n: 8 ;id: 7270.87_Ehrenbergina_glabra 7270.85_Ehrenbergina_glabra 7270.86_Ehrenbergina_glabra 7290.90_Ehrenbergina_glabra 7290.91_Ehrenbergina_glabra 7541.93_Ehrenbergina_glabra 7541.95_Ehrenbergina_glabra 7541.94_Ehrenbergina_glabra

**Step 2. Results of four ABGD analyses on subsets of sequences classified as included in four distinct genera.**

ABGD results for *Cassidulina*

Initial Partition with prior maximal distance P=1.67e-03

Distance JC69 Jukes-Cantor MinSlope=1.500000

Download (left click and save) or see below the tree file corresponding to this partition: click here

Group[ 1 ] n: 5 ;id: 17297.2_C.laevigata_BF20 17297.3_C.laevigata_BF20 17108.1_C.carinata_BF14 17108.2_C.carinata_BF14 17108.3_C.carinata_BF14

Group[ 2 ] n: 6 ;id: 20603.1_Cassidulina_SG 20603.2_Cassidulina_SG 20654_Cassidulina_FK 20645_Cassidulina_FK 20646_Cassidulina_FK 20648_Cassidulina_FK

Group[ 3 ] n: 18 ;id: 8124.1_Cassidulina_ADM 8124.2_Cassidulina_ADM 17113.1_Cassidulina_BF07/ 17113.2_Cassidulina_BF07/ 17076.1_Cassidulina_BF07/ 17076.2_Cassidulina_BF07/ 17076.3_Cassidulina_BF07/ 17082.1_Cassidulina_BF36 17157.2_Cassidulina_BF50 7642.1_Cassidulina_USH 17297.1_C.laevigata_BF20 17091.1_C.carinata_BF19 17091.3_C.carinata_BF19 17082.2_Cassidulina_BF36 17088.1_Cassidulina_BF50 17088.3_Cassidulina_BF50 17156.3_Cassidulina_BF50 17112_C.crassa_BF55

Group[ 4 ] n: 8 ;id: 20642_Cassidulina_FK 20643_Cassidulina_FK 20652_Cassidulina_FK 20655_Cassidulina_FK 20656_Cassidulina_FK 20657_Cassidulina_FK 20604.1_Cassidulina_SG 20653_Cassidulina_FK

ABGD results for *Cassidulinoides*

Initial Partition with prior maximal distance P=1.67e-03

Distance JC69 Jukes-Cantor MinSlope=1.500000

Download (left click and save) or see below the tree file corresponding to this partition: click here

Group[ 1 ] n: 26 ;id: 8068a_C.parkerianus_ADM 8227a_C.parkerianus_ADM 7828a_C.parkerianus_ADM 8182a_C.parkerianus_ADM 8068b_C.parkerianus_ADM 7795a_C.parkerianus_ADM 8182b_C.parkerianus_ADM 7828b_C.parkerianus_ADM 7313.3_Cassidulinoides_McM 14178.1_Cassidulinoides 14392.1_Cassidulinoides 14231.1_Cassidulinoides 7313.4_Cassidulinoides 3924a_C.parkerianus_TB 14178.2_Cassidulinoides 14231.2_Cassidulinoides 18356.3_Cassidulionides_large_RS 14392.4_Cassidulinoides 14231.3_ Cassidulinoides 14231.4_Cassidulinoides 18365.2_Cassidulinoides_RS 18365.1_Cassidulinoides_RS 18356.5_Cassidulinoides_large_RS 7313.1_Cassidulinoides_McM 3924b_C.parkerianus_TB_DQ452689 18356.2_Cassidulinoides_large_RS

Group[ 2 ] n: 14 ;id: 20624.1_Cassidulinoides_SG 20624.2_Cassidulinoides_SG 20627.1_Cassidulinoides_SG 20627.2_Cassidulinoides_SG 20638.1_Cassidulinoides_FK 20639.1_Cassidulinoides_FK 20639.2_Cassidulinoides_FK 20608_Cassidulinoides_parkeriensis_SG 20610_Cassidulinoides_parkeriensis_SG 20615_Cassidulinoides_SG 8027b_C.porrectus_ADM 8027a_C.porrectus_ADM 8299b_C.porrectus_ADM 8299a_C.porrectus_ADM

Group[ 3 ] n: 2 ;id: 20613_Cassidulinoides_parkeriensis_SG 20614_Cassidulinoides_parkeriensis_SG

Group[ 4 ] n: 22 ;id: 17120.1_G.subglobosa_BF11B 17120.2_G.subglobosa_BF11B 17128.3_C.parkerianus_BF 17128.2_C.parkerianus_BF 17128.4_C.parkerianus_BF 17124.4_C.parkerianus_BF 17133.2_C.parkerianus_BF 17124.1_C.parkerianus_BF 17141.1_C.parkerianus_BF 17132.1_C.parkerianus_BF 17085.2_C.parkerianus_BF 17085.1_C.parkerianus_BF 17124.3_C.parkerianus_BF 17131.2_C.parkerianus_BF 17133.4_C.parkerianus_BF 17141.3_C.parkerianus_BF 17133.3_C.parkerianus_BF 17128.1_C.parkerianus_BF 17132.3_C.parkerianus_BF 17131.1_C.parkerianus_BF 17131.3_C.parkerianus_BF 17107.1_C.parkerianus_BF

ABGD results for *Ehrenbergina*

* ABGD (Automatic Barcod Gap Definition)

/* web version 02/21/19 - 04:23PM

Waiting for results...

Fasta Format detected

Jukes Cantor distance done JC Partition 1 : found 1 groups (prior maximal distance P= 0.001000)

Only one partition found with your data. Please rerun with a lower X

Nothing to output

**End of program**

*ABGD results for Globocassidulina*

Initial Partition with prior maximal distance P=1.67e-03

Distance JC69 Jukes-Cantor MinSlope=1.500000

Download (left click and save) or see below the tree file corresponding to this partition: click here

Group[ 1 ] n: 31 ;id: 18344.1_Globocassidulina_RS 18362.1_Globocassidulina_RS 18362.2_Globocassidulina_RS 18375.1_Globocassidulina_RS 18375.2_Globocassidulina_RS 18375.3_Globocassidulina_RS 18377.4_Globocassidulina_RS 7411.1_G.subglobosa_NH 7411.2_G.subglobosa_NH 7411.3_G.subglobosa_NH 7400.2_G.subglobosa_NH 7400.3_G.subglobosa_NH 18300.1_G.subglobosa_RS 18302.2_G.subglobosa_RS 18308.2_G.subglobosa_RS 18305.3_G.subglobosa_RS 18299.3_G.subglobosa_RS 18309.3_G.subglobosa_RS 18309.2_G.subglobosa_RS 18309.1_G.subglobosa_RS 8234.206 8234.207 17213.1_Globocassidulina_17213.2_Globocassidulina_ 17213.3_Globocassidulina_ 8251.3_G.subglobosa_ADM 8251.2_G.subglobosa_ADM 8251.1_G.subglobosa_ADM 8125.3_Globocassidulina_ADM 8125.2_Globocassidulina_ADM 8125.1_Globocassidulina_ADM

Group[ 2 ] n: 1 ;id: 18362.3_Globocassidulina_RS

Group[ 3 ] n: 32 ;id: 7905_G.biora_ADM 7931_G.biora_ADM 7964_G.biora_ADM 7907.27_G.biora_ADM 7902.15_G.biora_ADM 7907.28_G.biora_ADM 7909.31_G.biora_ADM 7909.33_G.biora_ADM 7962.21_G.biora_ADM 7962.23_G.biora_ADM 7963.39_G.biora_ADM 1098.4_G.biora_NH 1099.4_G.biora_NH 1098.2_G.biora_NH 1099.1 1098.1 1098.3 7309.1_G.biora_NH 7309.3_G.biora_NH 7310.1_G.biora_NH 7310.2_G.biora_NH 17197.1_G.biora_ROT 17197.2_G.biora_ROT 17187.3_G.biora_ROT 17187.2_G.biora_ROT 17187.1_G.biora_ROT 17225.1_Globocassidulina_biora 17225.2_Globocassidulina_biora 14165.3_Globocassidulina_biora 14165.1_Globocassidulina_biora 14194.2_Globocassidulina_biora 14194.3_Globocassidulina_biora

Group[ 4 ] n: 14 ;id: 20618.1_Globocassidulina_SG 20623.1_Globocassidulina_SG 20628.2_Globocassidulina_SG 20623.2_Globocassidulina_SG 20620.1_Globocassidulina_SG 20620.2_Globocassidulina_SG 20621.1_Globocassidulina_SG 20621.2_Globocassidulina_SG 20617.1_Globocassidulina_SG 17063.1_G.minuta_BF11B 17063.3_G.minuta_BF11B 17078.2_G.rosensis_BF11B 17078.3_G.rosensis_BF11B 17121.3_G.rosensis_BF11B

Group[ 5 ] n: 1 ;id: 20628.1_Globocassidulina_SG

Group[ 6 ] n: 1 ;id: 17117.3_G.rosensis_BF11B

Group[ 7 ] n: 3 ;id: 17106.3_G.rosensis_BF11B 17106.2_G.rosensis_BF11B 17106.1_G.rosensis_BF11B

Group[ 8 ] n: 1 ;id: 17117.1_G.rosensis_BF11B

Group[ 9 ] n: 1 ;id: 17117.2_G.rosensis_BF11B
