# Supplementary material for: Cenozoic climatic changes drive evolution and dispersal of coastal benthic foraminifera in the Southern Ocean
Source: Sci Rep. 2021 Oct 6;11:19869. doi: 10.1038/s41598-021-99155-6 (PMC8494791; doi:10.1038/s41598-021-99155-6)
Supplement: Supplementary file 3 — Supplementary Information 3. [file 41598_2021_99155_MOESM3_ESM.pdf]

Appendix 3. SEM images of Southern Ocean Cassidulinidae.

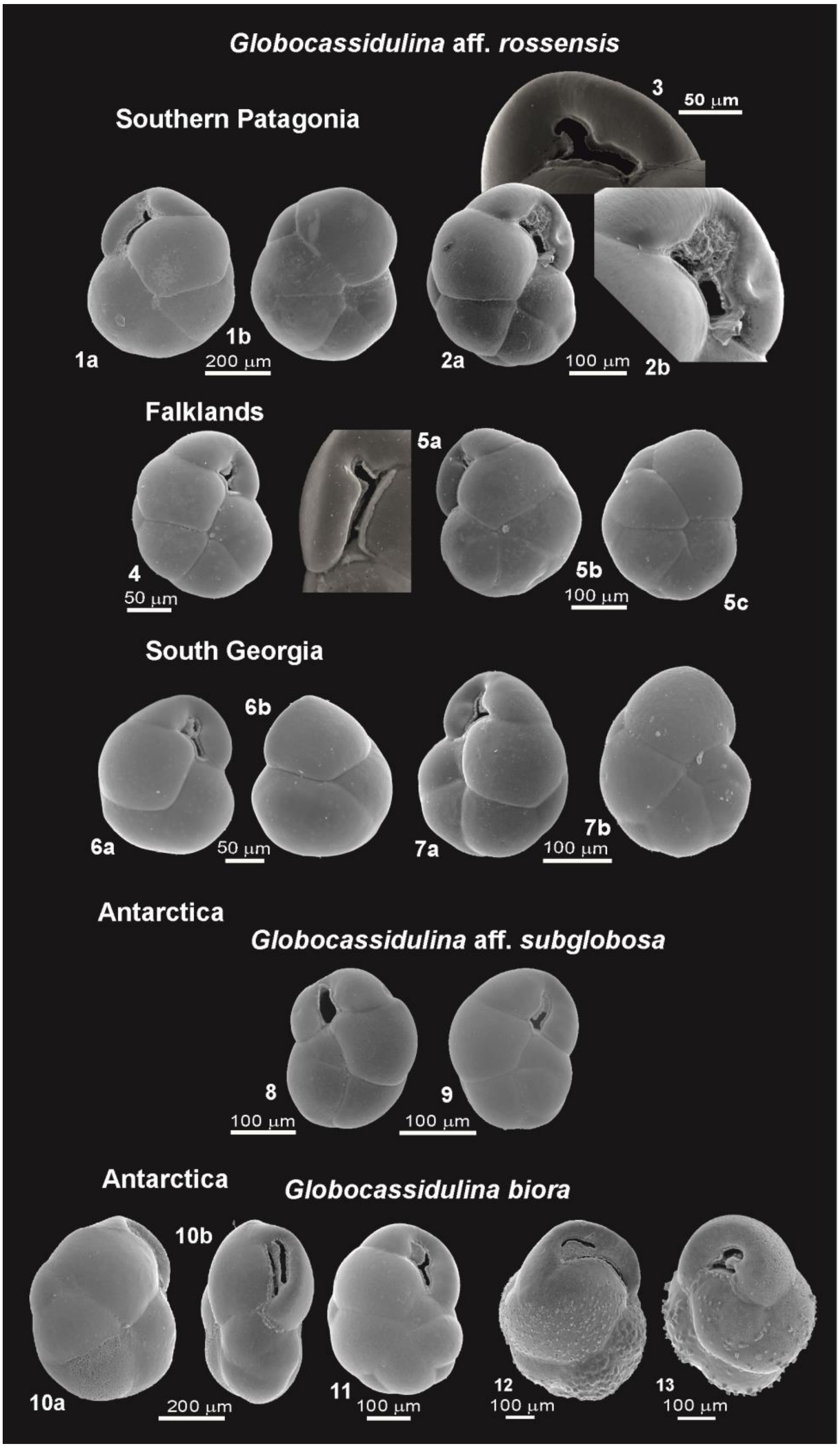

Plate 1. *Globocassidulina* aff. *rossensis* from southern Patagonia (**1** from site BF42, **2** and **3** from BF11B), the Falklands (**4** and **5** from Fk2), and South Georgia (**6** from SG27, **7** from SG17); *Globocassidulina* aff. *subglobosa* from the Ross Sea Antarctica (**8** from G1, **9** from BC1), *Globocassidulina biora* from South Shetlands (**10** and **11** after Majewski 2005) and its pustulose morphotype from the Ross Sea (**12** and **13** after Majewski et al. 2019). Specimen 2 corresponds to isolate 17078, 8 to 18362, and 9 to 18375. For detailed sample locations refer to Appendix 1.

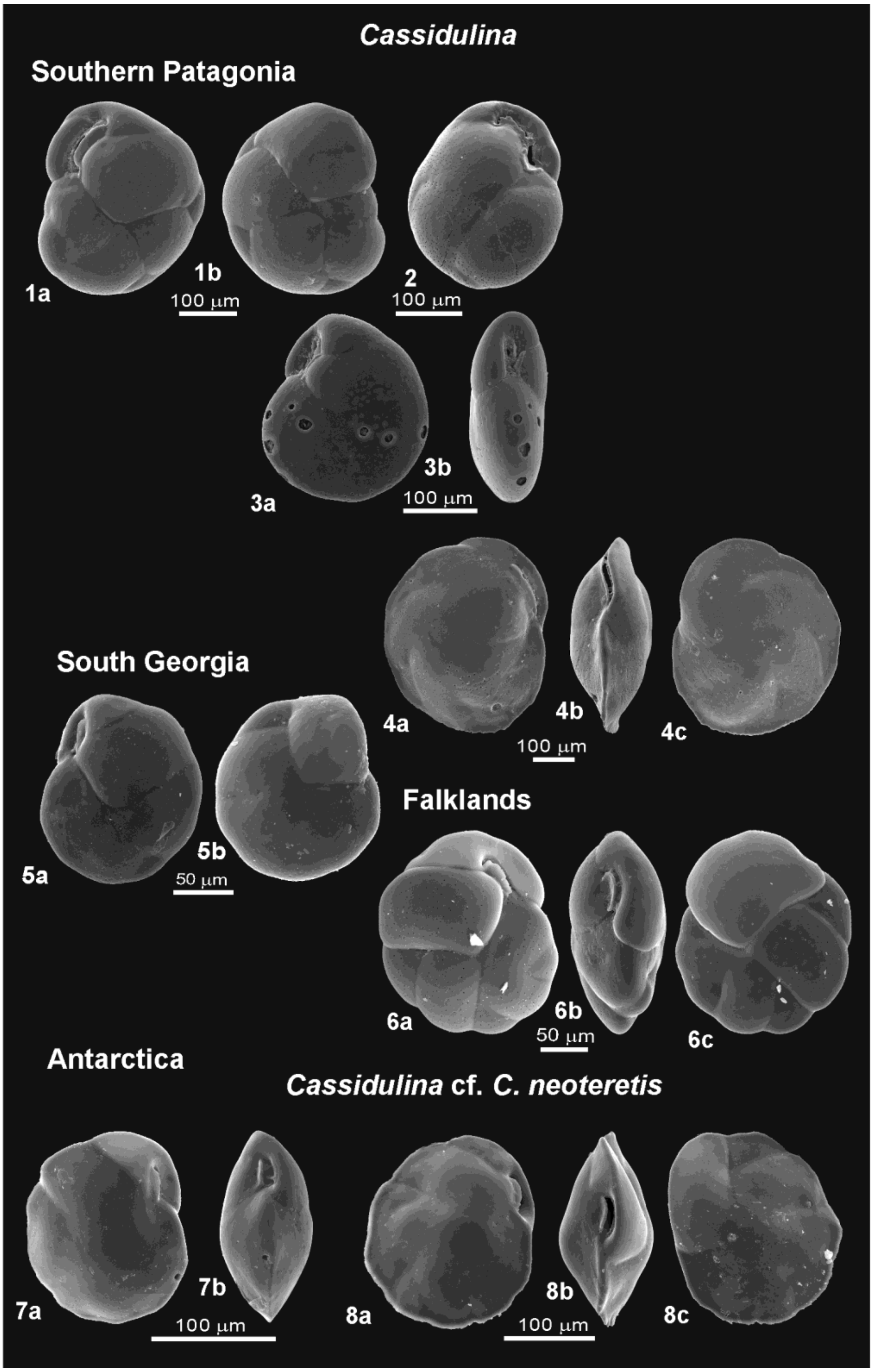

Plate 2. Moderately inflated to lenticular forms of *Cassidulina* from southern Patagonia (**1** from BF42, **2** from BF50 corresponds to isolate 17088, **3** from BF39, **4** from BF13; 1, 3 and 4 after Korsun et al, submitted), South Georgia (**5** from site SG12) the Falklands (**6** from Fk2), and *Cassidulina* cf. *C. neoteretis* from the Ross Sea (**7** and **8** after Majewski *et al.* 2020). It is unlikely that *Cassidulina neoteretis* (Kilfeather *et al.* 2011; Majewski *et al.* 2020) and other lenticular Cassidulinidae reported from Antarctica (Kennett 1968; Mead 1985; Mackensen and Douglas 1989; Mackensen *et al.* 1990, 1993; Melis and Salvi 2009) correspond to the only Antarctic isolate 8124 from Admiralty Bay, as lenticular forms were not reported from South Shetlands so far (Finger and Lipps 1981; Majewski 2005; Rodriguez *et al.* 2010). For detailed sample locations refer to Appendix 1.

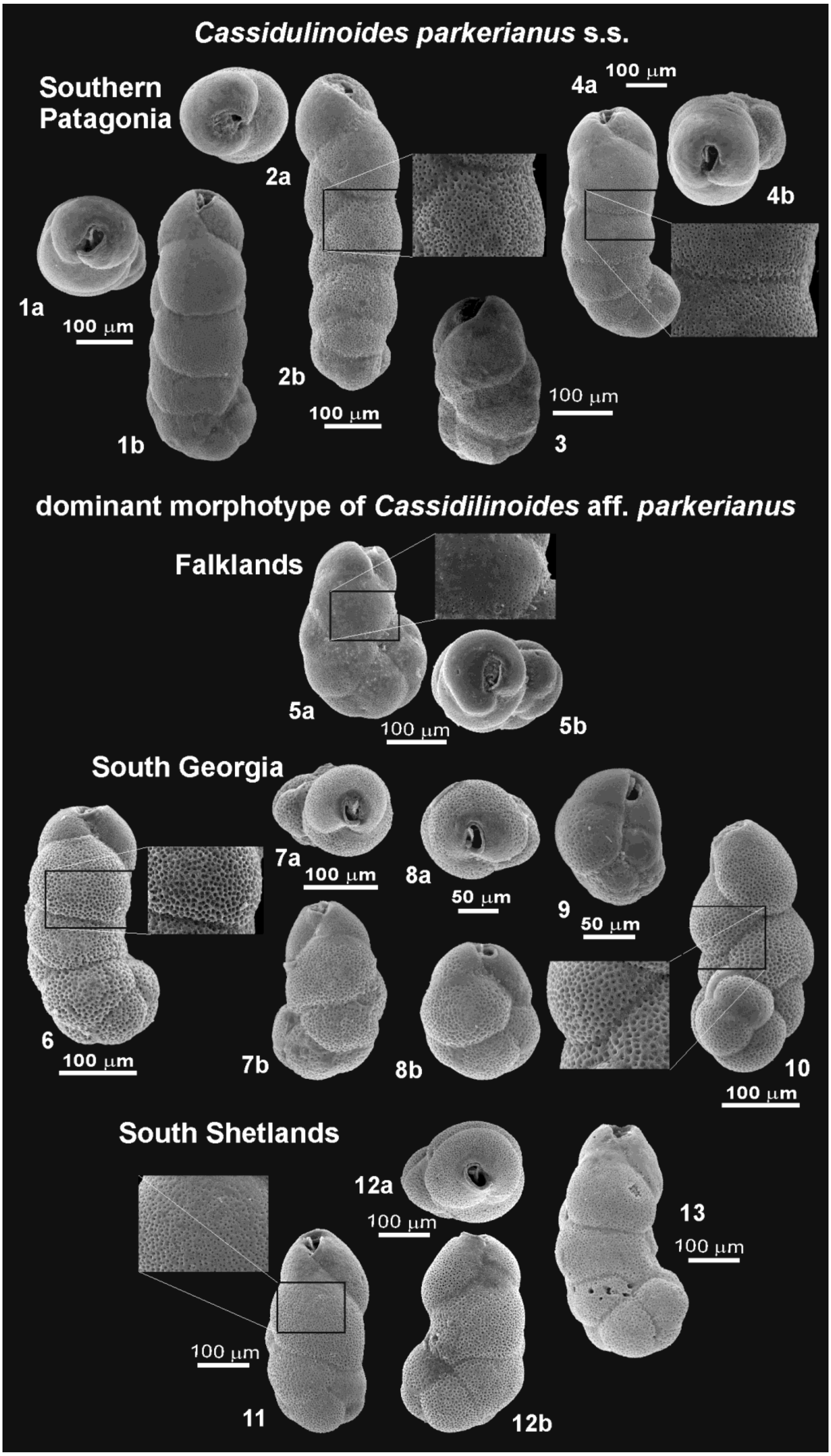

Plate 3. *Cassidulinoides parkerianus* s.s. from southern Patagonia (**1** from sample BF50, **2** from BF13, **3** and **4** from BF50), and morphologically alike specimens of dominant morphotype of *Cassidulinoides* aff. *parkerianus* from the Falklands (**5** from Fk2), South Georgia (**6** from SG17, **7** and **8** from SG21, **9** from SG21), including atypical specimen with depressed sutures (**10** from SG20), and from South Shetlands (**11**

to **13** after Majewski 2005). Specimen 3 corresponds to isolate 17085. For detailed sample locations refer to Appendix 1.

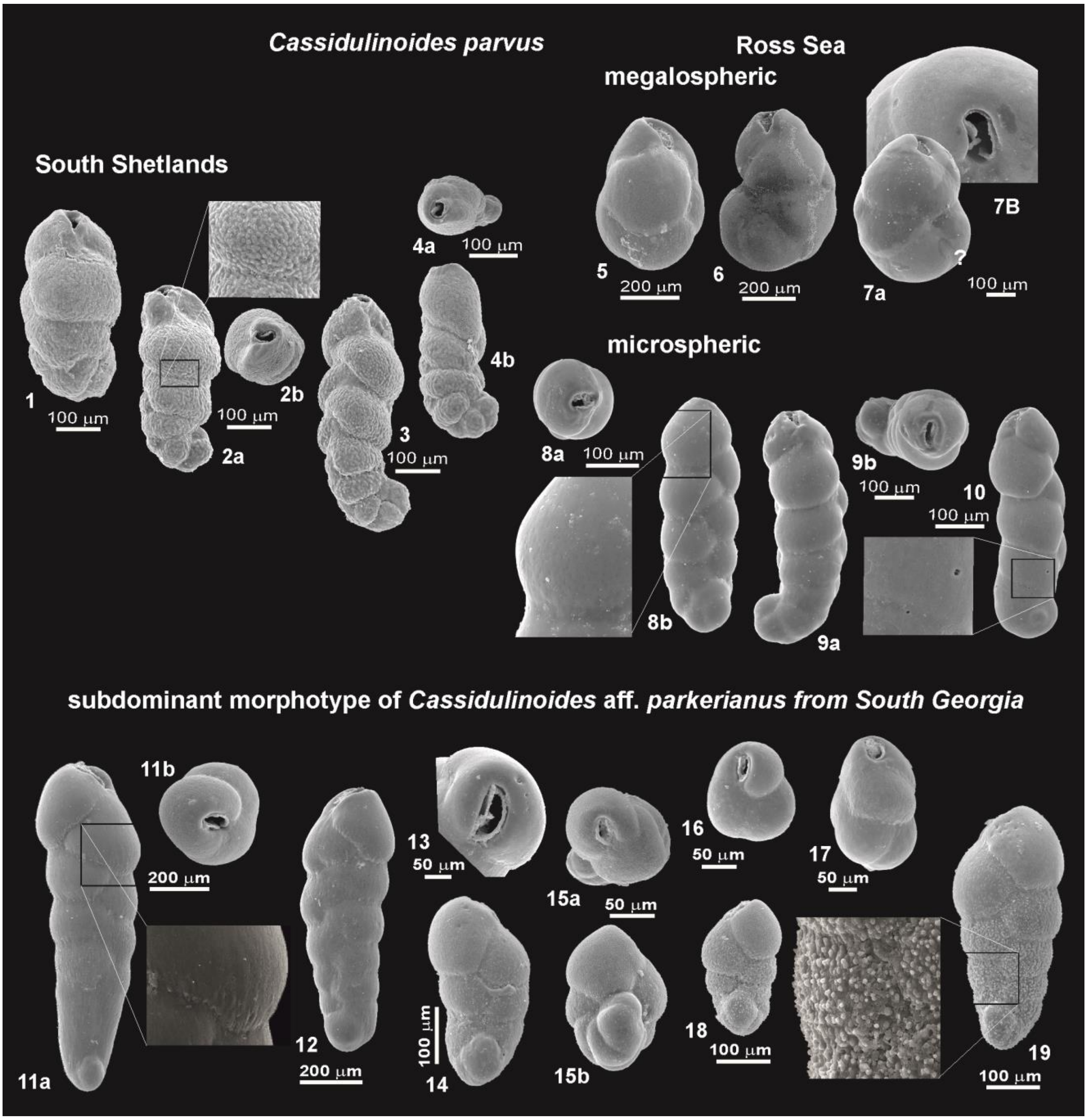

Plate 4. *Cassidulinoides parvus*, granular morphotype from South Shetlands (**1** to **4** after Majewski 2005), megalospheric (**5** and **6** from G4, **7** from Hut Point) and microsphelic variants of the smoothly-walled tubular morphotype from the Ross Sea (**8** to **10** from Cape Barn) as well as the subdominant smoothly-walled conical morphotype of *Cassidulinoides* aff. *parkerianus* from the South Georgia (**11** from SG9, **12** to **15** from SG14, **16** from SG14, **17** from SG27) and forms with granulate wall-texture (**18** from and **19** from SG09). Specimen 5 corresponds to isolate 18356. For detailed sample locations refer to Appendix 1.
