# Supplementary material for: Cenozoic climatic changes drive evolution and dispersal of coastal benthic foraminifera in the Southern Ocean
Source: Sci Rep. 2021 Oct 6;11:19869. doi: 10.1038/s41598-021-99155-6 (PMC8494791; doi:10.1038/s41598-021-99155-6)
Supplement: Supplementary file 4 — Supplementary Information 4. [file 41598_2021_99155_MOESM4_ESM.docx]

**Appendix 4.** Species nomenclature.

*Globocassidulina* *biora* was first described from the Vestfold Hills (East Antarctica) by Crespin (1960) and corresponds well with our specimens, which in the adult stage have a double aperture (Fig. 3.5). Our second Antarctic *Globocassidulina* species (Fig. 3.6), which has a single aperture oriented nearly perpendicular to the last suture, corresponds morphologically with *Globocassidulina* aff. *subglobosa*. It has long been established in the literature as an Antarctic species differing from *G. biora* (e.g., Osterman and Kellogg 1979; Finger and Lipps 1981; Ishman and Domack 1994; Violanti 1996; Melis and Salvi 2009; Gooday et al. 2014; Majewski et al. 2018, 2020), although the distribution of the two species (Fig. 1) and the test morphology of juvenile specimens may overlap. Molecular characterization is therefore important to correctly identify both species.

*Globocassidulina subglobosa* was originally described by Brady (1881); the locality was not specified but the type material in the Natural History Museum, London, is from *Challenger* Station 120, at 1225 m depth off Brazil. Brady (1884) records *G. subglobosa* from all oceans across a depth range from 22 to 5395 m, but notes that it is a predominantly deep-water species. It is regarded as an important abyssal species (Corliss 1979), although the abundance may be strongly overestimated, as it can be easily confused with immature forms of other Cassidulinidae species (Heron-Allen and Earland 1932; Nomura 1984). We analyzed deep-sea specimens identified as *G. subglobosa* from the abyssal eastern Pacific genetically. The sequences (unpublished) are very different from those of the Antarctic population. We cannot exclude the possibility that another genetically different species is present in the deep Southern Ocean. However, the case of the rotaliid genus *Epistominella*, in which the abyssal *E. exigua* clearly differs genetically from its Antarctic sister species *E. vitrea*, despite the latter having a bathymetric range of more than 1000 m (Pawlowski et al. 2007), strongly supports the possibility that the abyssal *G. subglobosa* is different from the Antarctic *G.* aff. *subglobosa*.

The sub-Antarctic species of *Globocassidulina* shows the bifurcated aperture and the characteristic lip that resemble these features in *G. rossensis* (Fig. 3.4). It was described as a sub-species of *Cassidulina crassa* by Kennett (1967) from the Ross Sea, Antarctica, and was later interpreted as an immature form of *G. biora* (Nomura 1983; Majewski and Pawlowski 2010). Because our sub-Antarctic species is genetically divergent from Antarctic *Globocassidulina*, it is regarded in the present paper as *G.* aff. *rossensis.*

Most of our specimens of *Cassidulinoides* were assigned upon collection to *C. parkerianus*, which has been considered to be a widely distributed species, reported from Antarctica, including the Ross Sea (Kennett 1968; Osterman and Kellogg 1979; Ward et al. 1987; Violanti 1996) and South Shetlands (Finger and Lipps 1981; Ishman and Domack 1994; Chang and Yoon 1995; Majewski 2005), as well as from South Georgia (Dejardin et al. 2018), Chilean Patagonia (Thompson 1978; Hromic et al. 2006; Hromic 2009; Arellano et al. 2011), the South Atlantic east of Patagonia and around the Falklands (Heron-Allen and Earland 1932). The species was originally described as *Cassidulina parkeriana* by Brady (1881); again, the locality was not specified but according to Brady (1884) it is confined to three Challenger stations spanning the depth range 82 to 320 m to the west of Patagonia. Our delimitation analyses suggest the presence of three species (Fig. 3), with the one limited to Patagonian fjords regarded as *C. parkerianus* s.s. The second, ranging from the Falkland Islands, South Georgia, and South Shetlands, which is the dominant morphotype and seems to be indistinguishable from its Patagonian counterpart, is regarded as *C.* aff. *parkerianus.* A sub-dominant morphotype of this species was noted so far only in South Georgia, where it was assigned by Heron-Allen and Earland (1929) and Earland (1933) to *Ehrenbergina crassa*.

A third species, currently reported only from Antarctica, is assigned to *Cassidulinoides parvus*. There is a clear genetic (Fig. 5) and morphologic distinction between populations sampled from the South Shetlands and those from the Ross Sea. Because there are no intervening samples, it is not known yet whether their ranges overlap. Individuals from the South Shetlands are smaller and more strongly elongated, and their adult chambers are less globular than the Ross Sea variant (Fig. 3.14). Pores are located on tiny projections, causing the test wall to appear hispid. In general, the test shape and chamber arrangement of this South Shetlands form are similar to *C. parvus* sensu Arellano et al. (2011) from Patagonia. The species was originally described as *Ehrenbergina parva* from South Shetlands as well as the broader Antarctic Peninsula region (Earland 1934). The type specimens show “increasingly inflated chambers towards the aperture”, which corresponds well with our specimens. Although the presence of the projections was not mentioned in the original description, the granular appearance of the test wall was noted but attributed to “coarse perforation”. Given the morphological similarities of our specimens to *E. parva,* as described by Earland (1934), and the same geographical distribution of Earland’s specimens and our material, we assigned our specimens to *C. parvus*.

Specimens from the Ross Sea, which are also identified as *C. parvus* based on molecular data, have thick, smooth, finely-perforated test walls and relatively few large chambers (Fig. 3.15). Only the last few chambers uncoil (Fig. 3.15). All *C. parvus* sequences from the Ross Sea analyzed in this study have been obtained from this morphotype. However, there are also much slenderer, strongly elongated forms present in the Ross Sea (Ward 1984), see Fig. 3.16. These have the same smooth test wall with minute pores and could be regarded as microspheric variants of the more massive individuals. A corresponding range of morphologic variability has been described for *C. parkerianus* (Brady 1881 in Jones 1994; Heron-Allen and Earland 1932).

**References**

Arellano, F., Quezada, L. & Olave, C. Familia Cassidulinidae (Protozoa: Foraminiferida) en canales y fiordos patagónicos chilenos. *An. Inst. Patagon.* **39**, 47–65 (2011).

Brady, H. B. Notes on some of the Reticularian Rhizopoda of the "Challenger" Expedition. Part III. *Q. J. Microsc. Sci.* **21**, 31–71 (1881).

Brady, H. B. Report on the Foraminifera dredged by H.M.S. Challenger during the Years 1873–1876. Report on the Scientific Results of the Voyage of H.M.S. Challenger during the years 1873–76. *Zoology* **9**, 1–115 (1884).

Chang, S. K. & Yoon H. I. Foraminiferal assemblages from bottom sediments at Marian Cove, South Shetland Islands, West Antarctica. *Mar. Micropaleontol.* **26**, 223–232 (1995).

Corliss, B. Size variation in the deep-sea benthonic foraminifer *Globocassidulina subglobosa* (Brady) in the Southeast Indian Ocean. *J. Foraminiferal Res.* **9**, 50–60 (1979).

Crespin, I. Some recent foraminifera from Vestfold Hills, Antarctica. *Rep. Tahoku Univ. Spec. Vol. Ser. 2* **4**, 19–31 (1960).

Dejardin, R. *et al.* “Live” stained) benthic foraminiferal living depths, stable isotopes, and taxonomy offshore South Georgia, Southern Ocean: implications for calcification depths. *J. Micropalaeontol.* **37**, 25–71 (2018).

Earland, A. Foraminifera, Part II, South Georgia. *Discov. Rep.* **7**, 27–138 (1933).

Earland, A. Foraminifera. Part III. The Falklands sector of the Antarctic (excluding South Georgia). *Discov. Rep.* **10**, 1–208 (1934).

Finger L. F. & Lipps J. H. Foraminiferal decimation and repopulation in an active volcanic caldera, Deception Island, Antarctica. *Micropaleontology* **27**, 11–139 (1981).

Gooday, A. J., Rothe, N., Bowser, S. S. & Pawlowski, J. Benthic foraminifera. *Biogeographic atlas of the Southern Ocean* (ed. De Broyer, C. *et al.*) 74–82 (SCAR Publications, 2014).

Heron-Allen, E. & Earland, A. Some new foraminifera from the South Atlantic, 2. *J. Roy. Micr. Soc.* **49**, 324–334 (1929).

Heron-Allen, E. & Earland, A. Foraminifera. Part I. The ice-free area of the Falkland Islands and adjacent seas. *Discov. Rep.* **4**, 291–460 (1932).

Hromic, T. Distribución batimétrica de foraminíferos bentónicos (Protozoa: Foraminiferida) al sur del estrecho de Magallanes (52–56 S), Chile. *An. Inst. Patagon.* **37**, 23–38 (2009).

Hromic, T., Ishman, S., & Silva, N. Benthic foraminiferal distributions in Chilean fjords: 47 S to 54 S. *Mar. Micropaleontol.* **59**, 115–134 (2006).

Ishman, S. E. & Domack, E. W. Oceanographic controls on benthic foraminifers from the Bellingshausen margin of the Antarctic Peninsula. *Mar. Micropaleontol.* **24**, 119–155 (1994).

Jones, R.W. *The Challenger Foraminifera* (Oxford University Press, 1994).

Kennett, J. P. New Foraminifera from the Ross Sea, Antarctica. *Cont. Cushman Found. Foram. Res.* **18**, 133–135 (1967).

Kennett, J. P. The fauna of the Ross Sea. Part 6. Ecology and distribution of the Foraminifera. *New Zealand DSIR Bull.* **186**, 46 (1968).

Majewski, W. Benthic foraminiferal communities: distribution and ecology in Admiralty Bay, King George Island, West Antarctica. *Pol. Polar Res.* **26**, 159–214 (2005).

Majewski, W. & Pawlowski, J. Morphologic and molecular diversity of the foraminiferal genus *Globocassidulina* in Admiralty Bay, West Antarctica. *Antarct. Sci.* **22**, 271–281 (2010).

Majewski, W., Bart, P. J. & McGlannan, A. J. Foraminiferal assemblages from ice-proximal paleo-settings in the Whales Deep Basin, eastern Ross Sea, Antarctica*. Palaeogeogr. Palaeoclimatol. Palaeoecol.* **493**, 64–81 (2018).

Majewski, W., Prothro, L. O., Simkins, L. M., Demianiuk, E. J. & Anderson J. B. Foraminiferal patterns in deglacial sediment in the western Ross Sea, Antarctica: Life near grounding lines. *Paleoceanogr. Paleoclimatol.* **35**, e2019PA003716 (2020).

Melis, R. & Salvi, G. Late Quaternary foraminiferal assemblages from western Ross Sea (Antarctica) in relation to the main glacial and marine lithofacies. *Mar. Micropaleontol.* **70**, 39–53 (2009).

Nomura, R. Foraminifera from the raised beach deposits on the east coast of Lutzow–Holm Bay, Antarctica. *Mem. Natl. Inst. Polar Res.* **28**, 219–230 (1983).

Osterman, L. E. & Kellogg,T. B. Recent benthic foraminiferal distribution from the Ross Sea, Antarctica: relation to ecologic and oceanographic conditions. *J. Foraminiferal Res.* **9**, 250–269 (1979).

Pawlowski, J., Bowser, S. S. & Gooday, A. J. A note on the genetic similarity between shallow- and deep-water *Epistominella vitrea* (Foraminifera) in the Antarctic. *Deep-Sea Res. II* **54**, 1720–1726 (2007).

Thompson, L. Distribution of living benthic foraminifera, Isla de los Estados, Tierra del Fuego, Argentina. *J. Foraminiferal Res.* **8**, 241–257 (1978).

Ward B. L. Distribution of modern benthic foraminifera of McMurdo Sound, Antarctica. Victoria University of Wellington, New Zealand; 211 pp. (unpublished thesis) (1984).

Ward B. L., Barrett P. J. & Vella P. Distribution and ecology of benthic foraminifera in McMurdo Sound, Antarctica. *Palaeogeogr. Palaeoclimatol. Palaeoecol.* **58**, 139–153 (1987).

Violanti, D. Taxonomy and distribution of recent benthic foraminifers from Terra Nova Bay (Ross Sea, Antarctica), Oceanographic Campaign 1987/1988. *Palaeontogr. Ital.* **83**, 25–71 (1996).
