# Supplementary material for: Cenozoic climatic changes drive evolution and dispersal of coastal benthic foraminifera in the Southern Ocean
Source: Sci Rep. 2021 Oct 6;11:19869. doi: 10.1038/s41598-021-99155-6 (PMC8494791; doi:10.1038/s41598-021-99155-6)
Supplement: Supplementary file 5 — Supplementary Information 5. [file 41598_2021_99155_MOESM5_ESM.pdf]

Appendix 5. Genetic dissimilarity.

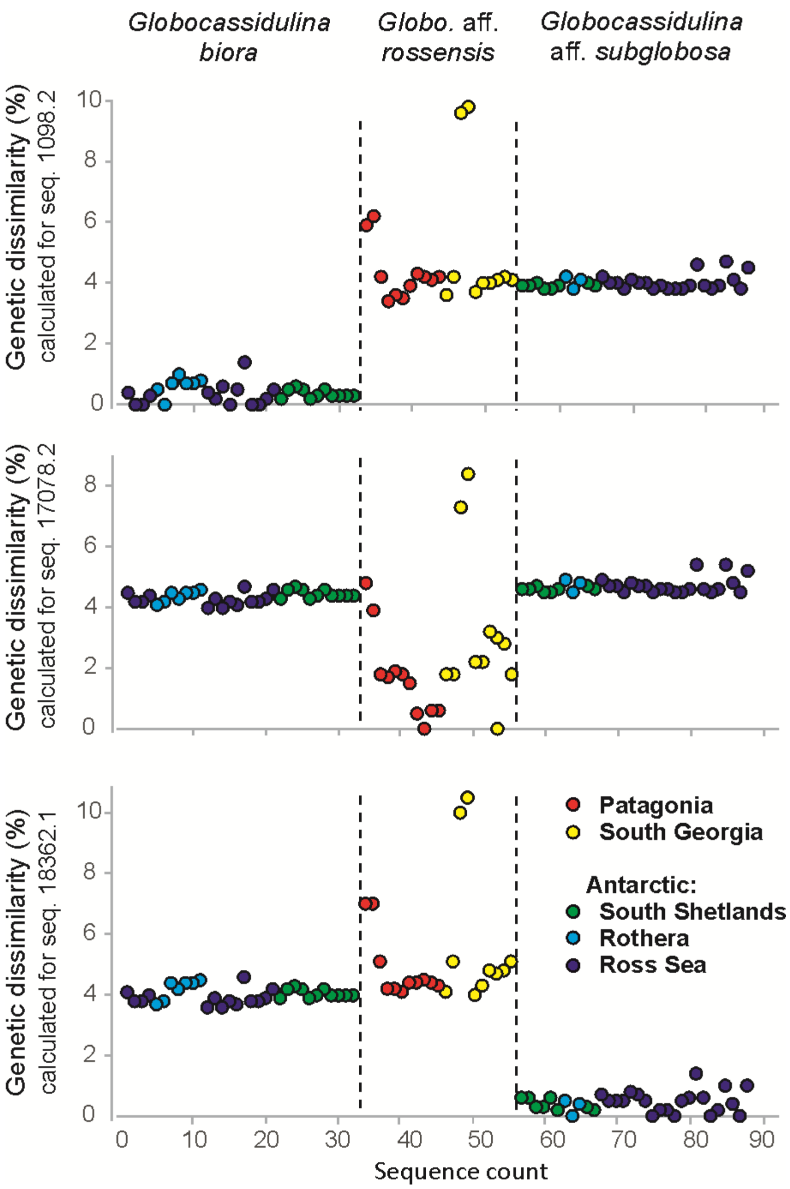

Genetic dissimilarity between different species of Southern Ocean *Globocassidulina* calculated for the most common/centrally located sequences in the haplotype networks shown on Fig. 4. Different colors represent different locations.

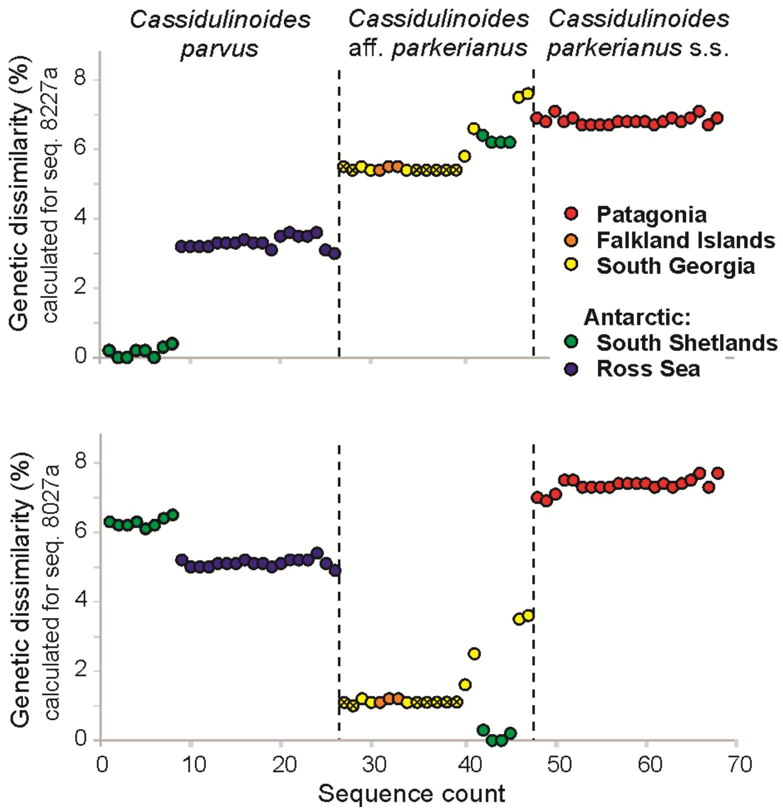

Genetic dissimilarity between different species of Southern Ocean *Cassidulinoides* calculated for the most common/centrally located sequences from Admiralty Bay (South Shetlands) in the haplotype networks shown on Fig 5. Different colors represent different locations. Crossed yellow circles indicate data obtained from the smoothly-walled, subdominant morphotype of *C.* aff. *parkerianus* from South Georgia.
