# Supplementary material for: Cenozoic climatic changes drive evolution and dispersal of coastal benthic foraminifera in the Southern Ocean
Source: Sci Rep. 2021 Oct 6;11:19869. doi: 10.1038/s41598-021-99155-6 (PMC8494791; doi:10.1038/s41598-021-99155-6)

**Appendix 6.** Maximum Likelihood phylogeny reconstruction using RAxML (see Material and Methods) based on the SSU rDNA. Bootstrap values are provided at the respective nodes.


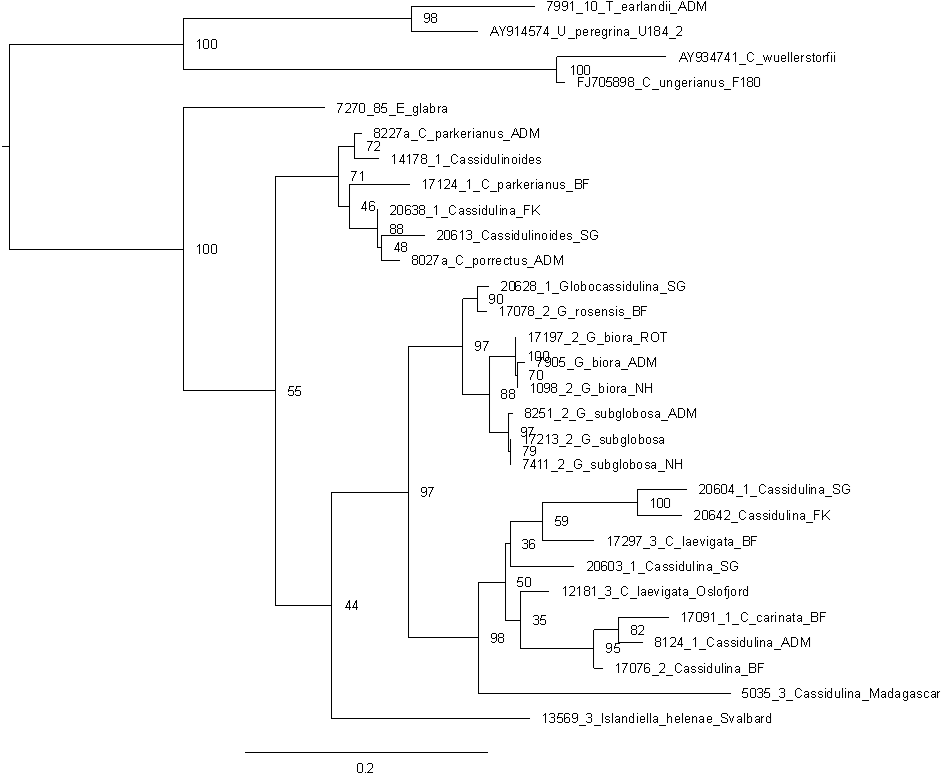

Supplement: Supplementary file 7 — Supplementary Information 7. [file 41598_2021_99155_MOESM7_ESM.docx]
